# Using brewing industry byproducts to create feed additives for aquaculture-raised rainbow trout under thermal stress

**DOI:** 10.1371/journal.pone.0360302

**Published:** 2026-11-03

**Authors:** Anna Rayne Layton, Jason D. Pough II, David D. Kuhn, Ann M. Stevens

**Affiliations:** 1 Department of Biological Sciences, Virginia Tech, Blacksburg, Virginia, United States of America; 2 Center for Emerging, Zoonotic, and Arthropod-borne Pathogens, Virginia Tech, Blacksburg, Virginia, United States of America; 3 Department of Food Science and Technology, Virginia Tech, Blacksburg, Virginia, United States of America; Tanta University Faculty of Agriculture, EGYPT

## Abstract

The aquaculture industry helps meet the seafood demands of a rapidly growing human population. The goal of this study was to determine the effects of feed additives, generated from byproducts of the beer brewing industry, on the growth rate, production parameters, and intestinal bacterial composition of aquaculture-raised rainbow trout (*Oncorhynchus mykiss*). Two prevalent brewery byproducts, brewer’s spent yeast (BSY) and brewer’s spent grain (BSG), were converted into high-value fish feed additives. To increase nutritional value of the byproducts, BSY was treated with exoenzymes, and BSG was used to cultivate black soldier fly (BSF; *Hermetia illucens*) larvae, which was treated with proprietary chitinases. During a seven-week trial using a recirculating aquaculture system, 22.15 ± 0.24 g (mean ± standard error) fish were randomly separated with 12 fish/tank in quadruplicate for each of the six diets (48 fish per diet), and tanks were randomly assigned diets. Survival rates were recorded daily, and fish were weighed every two weeks to track growth rates, adjust feed amounts, and calculate feed efficiency at the tank level. Fish intestinal mucosa swabs were collected at the beginning and end of the trial to analyze changes in the bacterial communities. Due to a malfunction in the chiller that maintained cool water temperatures, temperature and dissolved oxygen became substantial chronic (two week) and then acute (12–24 hr) stress factors near the end of the trial. Therefore, the research focus shifted to understand the impacts of thermal and oxygen stress on fish fed these novel feed additives. Clear changes in the structure of the bacterial intestinal community were observed in the fish between the start and end of the study as evaluated by 16S rRNA gene analysis. Results suggest that fish fed three of the experimental diets benefitted by having higher survival rates and growth rates when subjected to the unplanned stress.

## Introduction

Aquaculture, the cultivation of aquatic organisms in a controlled environment, is an important alternative to wild-caught fisheries that can be used to meet seafood demands of a growing human population. Fish are an important protein source for humans, providing nutrients including, but not limited to, omega-3 fatty acids, vitamin D_3_ and B_12_, and minerals such as iron and zinc [1]. In the past thirty years, the annual output of global aquaculture production has increased by 609 %, and fish consumption has doubled to over 20 kg per capita [2]. Rainbow trout (*Oncorhynchus mykiss*), a freshwater fish constituting 97% of farmed trout production globally, is one of the most popular farmed-fish species as they are easy to spawn, fast growing, and highly tolerant to a range of environmental conditions [3–5]. The reliance on aquaculture continues to grow rapidly, therefore, it is important to conduct research on methods that can improve efficiency, sustainability, food security, animal health and welfare in the industry [6]. The influence of nutrition on the fish intestinal bacterial community and overall animal health is one active area of research, including the use of sustainable feed additives.

Beer is one of the most popular beverages worldwide, but it poses various environmental challenges in the beer brewing industry, especially concerning waste production [7]. Two of the largest byproducts of the beer brewing process are brewer’s spent grain (BSG) and brewer’s spent yeast (BSY; *Saccharomyces cerevisiae*), consisting of approximately 85% and 15% of the waste, respectively [8]. BSG, produced after the mashing step of the brewing process, is available year-round at little to no cost [9] and composed mostly of fiber [10]. However, it can be used as a low-cost substrate to cultivate black soldier fly (BSF; *Hermetia illucens*) larvae. In return, BSF larvae convert the low-value waste product into a high-value aquaculture feed additive rich in lipids and protein [11–15]. BSY is another promising protein source and has been shown to be able to replace a portion of fishmeal in fish feed [16,17]. Historically, fishmeal is the main protein feed ingredient and it relies on wild-caught fish [18]. The primary obstacle in converting BSY into a high-value feed additive is the low digestibility of the yeast cells to the fish; this can be attributed to the high rigidity and low permeability of the cell wall of the yeast, which is made of various macromolecules including ß-glucans and chitin [19]. By further processing the BSY byproduct with extracellular enzymes, the bioavailability of nutrients to the fish can be significantly increased [20]. In addition to recycling food waste generated by the food industry, the use of BSY and BSF as feed additives in aquaculture may also provide important health benefits to the fish, such as having prebiotic effects, thereby serving as a viable alternative to the traditional application of antibiotics in aquaculture.

As the use of antibiotics remains prevalent across various industries including aquaculture, concerns about consumer safety, ecological impacts on the environment, and antibiotic resistance continue to grow, prompting producers to look for alternatives [21]. Antibiotics are known to reduce bacterial diversity and induce dysbiosis in the gastrointestinal (GI) tract, one of the main entry points for pathogens [22–24]. Large shifts in bacterial community composition have been shown to be a prominent risk factor for pathogen invasion [25]. As an alternative to prophylactic treatment by antibiotics, it has been proposed that modulation of the intestinal bacterial community of fish may help mitigate disease. As such, feed additives that might serve as prebiotics have been an active area of research [23,26].

Prebiotics refer to host non-digestible food ingredients that stimulate growth of beneficial intestinal bacteria, leading to improvements in the health of the host [27]. Commonly, prebiotics are thought to work by increasing the number of beneficial bacteria, therefore decreasing the risk of harmful bacteria proliferating due to factors such as competition for space and nutrients [28]. Research has shown that mannan oligosaccharides (MOS) used as a dietary supplement in rainbow trout feeds, derived from the cell wall of yeast, significantly reduced mortality rates and improved indicators of immune function [29]. In addition to MOS, various *in vivo* and *in vitro* studies have proved that nutrients such as ß-glucans [30,31], lipopolysaccharides [30,32], and yeast extract [33] can improve rainbow trout innate immune function. Both the indirect and direct effects of prebiotics on the bacterial community of the intestinal tract play an important role in host health.

The goal of this study was to determine the effects of BSY and BSF (cultured on BSG) used as feed additives on the growth rate, production parameters, and intestinal bacterial composition of rainbow trout. It was hypothesized that the feed additives introduced into the experimental diets would lead to increased fish growth and production parameters without causing intestinal dysbiosis. However, due to an unexpected malfunction in the chiller used to maintain cool water temperatures, thermal stress became an important factor towards the end of the study in the form of both chronic and acute stress events. During the trial, water temperature exceeded the optimum (14–16˚C) and suitable growth ranges (16–19˚C) of rainbow trout, and ultimately surpassed the temperature range for survival (10–25˚C) (S1 Fig) [34]. Results suggest that the feed additives in three diets appear to have had a positive impact on the overall fish health and survival.

## Materials and methods

### Fish and animal husbandry

Original experimental methods were approved by the Virginia Tech Institutional Animal Care and Use Committee (IACUC) under protocol #20-084. As per the IACUC protocol, fish were checked at least twice a day for welfare. Fish were removed from the study and euthanized, using a 250 mg/L MS-222 (tricaine methanesulfonate; Syndel, Ferndale, WA, USA) water bath buffered with equal parts sodium bicarbonate, if they exhibited severe anorexia, appeared abnormally stressed, or exhibited external signs of injury/infections [35]. However, an unanticipated adverse event occurred during the study due to a chiller malfunction that resulted in elevated water temperatures overnight. This acute thermal stress caused a high mortality rate of fish. All remaining animals were immediately and humanely euthanized and sampled; data was recorded and subsequently analyzed as described under the auspices of the submitted manuscript. The incident was reported to and recorded by the IACUC under the same protocol, and changes were made to the RAS for future work, including the addition of a second chiller and a temperature alarm system.

Juvenile rainbow trout, between four to five months of age, were acquired from the Wytheville State Fish Hatchery (Max Meadows, VA, USA) (Time point 0, T0). The fish were acclimated to a recirculating aquaculture system (RAS) using carbon-filtered municipal water for three weeks before beginning the experimental trial (Time point 1, T1). The cold-water RAS included 30 170-liter polyethylene tanks and was equipped with a sand filter and two bead filters for solids filtration and nitrification, and a chiller to maintain cool water temperature. Throughout the duration of the trial, the system was exposed to halogen overhead lighting on an approximate 14:10 hour (light:dark) photoperiod with no natural light. Water quality parameters monitored throughout the trial included temperature, pH, dissolved oxygen, total ammonia-N, nitrite-N, and nitrate-N [36]. Temperature and dissolved oxygen were measured every day, and the remaining parameters were measured every other day. During the three-week acclimation period, all fish were fed a commercial feed (Ziegler Bros., Finfish Silver Semi-Floating, Gardner, PA, USA). The commercial feed consisted of 40% crude protein, 10% crude fat, 4% crude fiber, 12% moisture, and 8% ash.

### Feed preparation

Experimental diets consisted of five treatment groups with the dietary supplement top coated on the commercial feed; the commercial feed was used as the control diet. Powdered yeast (i.e., BSY treated with exoenzymes) and insect products (i.e., BSF treated with proprietary chitinases) were individually resuspended in deionized water (dH_2_O) according to the desired feed additive concentration (see below) and placed into a polypropylene wash bottle (Thermo Fisher Scientific, Waltham, MA, USA). Commercial feed was loaded into a 100 L portable cement mixer (Central Machinery, Moses Lake, WA, USA) where the experimental products were then sprayed in intervals until distributed evenly onto the feed and left to tumble for a minimum of 15 min after all the liquid product was applied. The feed was left to dry at room temperature with a fan blowing across it for 24−48 hr before being stored at −20˚C for the remainder of the trial.

The powdered feed additives were resuspended in dH_2_O using the amount of dry product needed to reach the desired final percent concentration, based on the total mass of feed pellets used for each diet. Therefore, the final concentration refers to the percent concentration of the additives present on the coated commercial feed (w/w%). Exoenzyme-treated BSY was coated on the feed for two diets at a 2% and 0.75% concentration (BSY1 and BSY2, respectively). Additional experimental diets were coated with two different insect products (Maltento, Cape Town, South Africa), denoted “old” (from a previous trial [37],) and “new”, at a 3% and 0.75% concentration (BSF3 and BSF4, respectively). Both insect products consisted of BSF larvae cultured on BSG, which were processed using a proprietary method involving chitinase. Additionally, during the production of BSF4, the product was heated to over 80˚C. The last diet was a combination of BSY2 and the BSF4 insect product top coated at a 1.5% concentration (mixed at 0.75% for each of the diets, 1.5% total).

### Experimental design

After excluding exceptionally large or small fish, fish of average size, 22.15 ± 0.24 g (mean ± standard error), were randomly separated into 12 fish per tank two days before the start of the experimental trial, with four replicates for each of the six diets (48 fish per diet). In total, 24 of the 170-liter tanks were used, with diets randomly assigned to each. Survival rates were recorded daily, and fish were weighed every two weeks throughout the duration of the trial to track growth rates, adjust feed amounts, and calculate feed efficiency at the tank level. Fish were fed at a rate of 2.5% body weight per day at the tank level based on modeled linear growth over each two-week period during the entire trial.

Just prior to the start of the experimental diet trial (T1), eight fish were harvested directly from the RAS to collect controls for bacterial community analysis. The fish were humanely euthanized using 250 mg/L MS222 buffered with sodium bicarbonate as described above and dissected immediately. After approximately seven weeks (Time Point 2, T2), following an unforeseen acute stress event due to chiller malfunction, all remaining live fish were euthanized following the same procedure as T1. All T2 fish were separated by tank into large, plastic zipper-lock storage bags (SC Johnson, Racine, WI, USA) and stored at −20˚C for three days until the animal tissues could be processed. The day before a tissue harvest, fish were separated one-by-one into individual plastic zipper-lock storage bags (Food Lion, Salisbury, NC, USA; CVS, Woonsocket, RI, USA). Approximately nine hours prior to dissection, the fish were moved from −20˚C to 4˚C to thaw overnight.

Initially, eight T2 fish of approximately equal size were used to collect production data and obtain intestinal samples for bacterial community analysis for four of the six diets (Control, BSY2, BSF3, and Mix). Those three experimental diets were chosen based on the highest survival rate during the acute stress event. Approximately two weeks later, six additional euthanized fish from the original four diets, drawn randomly across tanks and not including those that died of natural causes, were used to collect production data only. In addition, for the production data from the remaining two diets (BSY1 and BSF4), eight euthanized fish of approximately equal size and another six, drawn randomly, were used to obtain production and biometric data consistency across all diets on the tank level (n = 14 total).

### Production data acquisition from euthanized fish and statistical analysis

Due to the unexpected chiller malfunction, the growth data (weight gain, feed conversion ratio [FCR]) presented in the results accounts for just six of the seven weeks of the trial. Week six is when the last planned tank-level weigh-in occurred prior to the large-scale unplanned mortality event at week seven. The biometric data (fillet yields, viscerosomatic index [VSI], hepatosomatic index [HSI], Fulton’s condition factor) were calculated after the harvest of week seven euthanized fish. The following equations were used for calculations:


$$\begin{array}{c} Weight\;gain\;(g)={Weight}_{Final\;Mean\;Weight\;}-{Weight}_{Initial\;Mean\;Weight\;} \end{array}$$

$$\begin{array}{c} Food\;Conversion\;Ratio\;(FCR)=\;\frac{Total\;Feed\;Fed\;(g)}{Weight\;gain\;(g)} \end{array}$$

$$\begin{array}{c} Fillet\;Weight\;(\%)=\text{1}\text{0}\text{0}\times \frac{Fillet\;Weight(g)}{\;Body\;Weight(g)} \end{array}$$

$$\begin{array}{c} Viscerosomatic\;Index\;(VSI)(\%)=\text{1}\text{0}\text{0}\times \;\frac{Weight\;of\;Viscera(g)}{Body\;Weight(g)} \end{array}$$

$$\begin{array}{c} Hepatosomatic\;Index\;(HSI)(\%)=\text{1}\text{0}\text{0}\times \frac{Weight\;of\;Liver\;(g)}{Body\;Weight\;(g)} \end{array}$$

$$\begin{array}{c} Fulton^{\prime }s\;Condition\;Factor=\text{1}\text{0}\text{0}\;x\;\frac{Body\;Weight\;(g)}{{\left[Body\;Length\;(cm)\right]}^{\text{3}}} \end{array}$$

During each of the tissue harvests, the fish were measured and dissected individually using ethanol flame-sterilized surgical tools. The dissection area of each fish (anus to gills) was first rinsed with sterile phosphate-buffered saline (PBS) for approximately five seconds. Between sampling of each fish, cutting boards, surgical tools, and gloves were all sanitized with 70% ethanol. The production factors measured were total weight, whole length (nose to end of caudal fin), body length (nose to beginning of caudal fin), viscera weight, liver weight, and fillet weight. From the production data, biometrics such as fillet yield, VSI, HSI, and condition factor were calculated using standard equations as described above [36].

Fish production data including weight gain, FCR, fillet yields, VSI, HSI, Fulton’s condition factor, and survival rates were all analyzed in Microsoft Excel (v.16.76.1, Redmond, Washington) using a one-way analysis of variance (ANOVA) test from the Analysis ToolPak add-in. No post-hoc testing was performed on production data since the ANOVA results indicated no statistically significant differences (P > 0.05) of any metrics.

### Bacterial community sampling

At T1, 16 intestinal swab (2/fish) and eight fecal (1/fish) samples from eight euthanized fish were collected to be used as controls during the bacterial community analysis of T2 samples. The same sampling procedure was followed at T2 for eight euthanized fish from four of the six diets (Control, BSY2, BSF3, and Mix) and for eight control fish that died naturally (ND). However, sufficient fecal material was unable to be recovered from the fish sampled at T2. For the fish used for bacterial community analysis sampling, a portion of the intestinal tract was removed, consisting mostly of the midgut and hindgut. The segment was then cut open lengthwise and laid flat to obtain two swabs of the mucosal layer. A nylon flocked swab, flexible mini-tip, in a sterile peel pouch was used for all swab samples (Copan Diagnostic, Murrieta, CA, USA). Half the intestinal tract was swabbed lengthwise while rotating the swab with gentle pressure, and the other half swabbed following the same procedure to obtain two swabs per fish. Each of the swabs were transferred individually into 1.5 ml polypropylene microcentrifuge tubes (USA Scientific, Ocala, FL, USA) and flash frozen in a 70% ethanol dry ice bath before short term storage at −20˚C and subsequent long-term storage at −80˚C after the completion of the harvest. Any fecal material present (T1 only) was recovered by using a metal spatula to gently scrape the material off the mucosa, which was then stored following the same conditions as the swabs.

DNA extractions were performed within a three-week period after each harvest. Water column samples were collected (~2.5 L each; n = 2) from the RAS at the start (T1) and end (T2) of the trial, stored at 4˚C, and processed within 24 hours using vacuum-filtration (Corning 0.22 µm filter, Corning, NY, USA). A sterile swab was used to scrape the filter retentate before being placed into a PowerBead tube (Qiagen, Germantown, MD, USA) and stored at −20˚C. A swab of the plastic storage bags, a sterile swab, and beads from the PowerBead tube were collected as negative controls. Lastly, samples of the control feed and each feed additive were collected in 15 ml conical tubes and then distributed by weight directly into four PowerBead tubes per each feed sample group (n = 4, 16 total). Sample weights for DNA extraction were as follows (mean ± SEM): control feed pellets (12.58 ± 0.77 mg), powder yeast product (11.93 ± 0.60 mg), “old” liquid insect product 1 (19.54 ± 3.46 mg), and “new” liquid insect product (19.09 ± 3.02 mg).

### DNA extraction

Genomic DNA (gDNA) was extracted from all samples using the Qiagen DNeasy PowerLyzer PowerSoil Kit following the manufacturer’s protocol with the following alterations: 10-min incubation at 72˚C after addition of C1 and addition of 50 µl of dH_2_O before the elution step including a 5-min incubation at 72˚C. Swabs, feces, and feed additive samples were added directly into the PowerBead tubes. After the extraction of DNA, sample quality was analyzed via total gDNA concentration and purity (A_260_/A_280_, A_260_/A_230_) using a nanospectophotometer (Implen, Westlake Village, CA, USA). Samples were stored at −20˚C until polymerase chain reaction (PCR) amplification.

### PCR amplification

The 16S rRNA gene V4 region was amplified via PCR in a thermal cycler (Bio-Rad Laboratories, Hercules, CA, USA) from each gDNA sample in triplicate with dH_2_O used as a negative control. A positive control of *Vibrio parahaemolyticus* RIMD 2210633 gDNA template at 50 ng/µl was used for each sample set. An experimental PCR reaction consisted of 12.5 µl of Q5 High-Fidelity 2X Master Mix (New England Biolabs, Ipswich, MA, USA), 1.25 µl of barcoded 515 forward and 1.25 µl of 806 reverse universal primers (500 nM; [38]), 120 ng of gDNA template, and a variable amount of dH_2_O to bring the final volume to 25 µl [39]. The universal primer concentration was doubled (1000 nM) for four mucosa samples (two from BSY2 and two from BSF1) where PCR was initially unsuccessful under the previously stated conditions. Additionally, the gDNA template was decreased in half (60 ng) for one fecal sample (Control, T1) for the same reason. PCR products could not be obtained from four fecal and two mucosal samples (Control, T1). The thermocycling conditions were as follows: initial denaturation at 98˚C for 30 sec, 30 cycles of denaturing at 98˚C for 10 sec, annealing at 55˚C for 20 sec, and elongation at 72˚C for 15 sec, as well as a final elongation at 72˚C for two min.

Following PCR, the triplicate products were combined, totaling 75 µl. An aliquot of 10 µl was visualized using gel electrophoresis on a 1% agarose gel and imaged (Bio-Rad ChemiDoc). The remaining 65 µl of PCR products were stored at 4˚C for short-term storage until being purified using the Qiagen QIAquick PCR Purification Kit following the manufacturer’s instructions with the following alterations: incubation at 72˚C for 5 min prior to elution with dH_2_O. The purified PCR products were subsequently stored at −20˚C for long-term storage before preparation for sequencing. A nanospectophotometer was used to evaluate the same parameters recorded for the initial DNA extractions. For further accuracy of purity and yield, amplicons were also analyzed via Qubit fluorometry at the Genomics Sequencing Center (GSC) of the Fralin Life Sciences Institute at Virginia Tech (Blacksburg, VA, USA).

### Sequencing

Purified V4 amplicons were pooled at a 10 nM concentration to be further processed, cleaned, and sequenced by the GSC. The pool was first filtered using Solid Phase Reversible Immobilization (SPRI) beads to remove unwanted DNA and to concentrate the V4 region amplicons. The amplicons were then purified again using Pippin Prep (Sage Science, Beverly, MA, USA) to remove contaminating host 18S rDNA and further select for the V4 target region. The processed pool was analyzed by TapeStation for quality control and then sequenced on the Illumina MiSeq platform using 500 cycles of 250 bp paired-end sequencing at a concentration of 10pM with phiX spiked in at 25%.

### Bioinformatics

Due to very low-quality reverse reads (Run 2) from the paired-end sequencing, only the forward reads (Run 1) were utilized in a single-read manner for bacterial community analysis. The Quantitative Insights Into Microbial Ecology (QIIME2 v. 2020.2 [40],) bioinformatics pipeline was used to analyze the generated amplicon sequences for bacterial diversity and identification. All steps using QIIME2 were performed using high-throughput computing clusters provided by the Advanced Research Computing (ARC) team at Virginia Tech (Blacksburg, VA, USA). DADA2 [41] was used to denoise the sequences and generate amplicon sequence variants (ASVs) and the sample-wise abundances of each ASV. The sequences were filtered using DADA2 to remove ASVs from eukaryotic-associated sequences (host DNA contamination) and low frequency (< 0.001% of total) ASVs [39]. The sequences were assigned taxonomy using a pre-trained 16S V4 region-specific classifier from the SILVA database, version 138.1 [42–44].

Filtered and taxonomically-assigned ASVs were then analyzed using R v.4.3.2 [45] with various packages including: qiime2R v.0.99.6 [46], phyloseq v.1.46.0 [47], vegan v.2.6-4 [48], ggplot2 v.3.4.4 [49], ComplexHeatmap v.2.18.0 [50], and dplyr v.1.14 [51]. Phylum-level bar plots were created using the 100 most abundant ASVs, representing over 99% of the total bacterial community of the mucosal samples from each treatment group. The same representation (99%) was true for all other sample groups except for the pellets of control feed (89%) and the T2 water column (96%) samples.

### Bacterial community statistical analyses

Alpha and beta diversity analyses were performed in R using files imported and generated from QIIME2. For beta diversity, the R packages phyloseq v.1.46.0 [47], vegan v.2.6-4 [48], and pairwiseAdonis v.0.4.1 [52] were used as well. Alpha diversity was analyzed using three metrics: Shannon diversity index, Pielou's evenness, and observed ASVs. The Shannon metric includes species abundance and evenness, Pielou’s evenness accounts for species diversity and richness, and observed ASVs is a count of the number of unique sequences present. Alpha diversity metrics were analyzed for statistically significant differences (P < 0.05) using the non-parametric Kruskal-Wallis test followed by Dunn’s post-hoc test with Bonferroni correction for pairwise comparisons.

Beta diversity non-metric multidimensional scaling (NMDS) plots were creating using the weighted and unweighted unique fraction (UniFrac) distance, which is a distance metric that accounts for phylogenetic information. The unweighted UniFrac distance only looks at the presence and absence of species, whereas the weighted UniFrac distance incorporates species abundance data as well. Statistical significance (P < 0.05) was calculated using the permutational multivariate analysis of variance (PERMANOVA) with 999 permutations, followed by a pairwise comparison with Bonferroni correction for multiple testing.

Differential abundance was determined on the family level using analysis of compositions of microbiomes with bias correction 2 (ANCOM-BC2) [53] from the R package ANCOMBC v.2.4.0 [54,55]. Significant differences (P < 0.05) in intestinal bacterial composition were determined using time or diet as the covariate of interest for multi-group comparisons. The Bonferroni correction was used to control for possible error caused by multiple testing. For multiple pairwise comparisons against a pre-specified group, the function within ANCOMBC for Dunnett’s type of test was used [56–58]. Dunnett’s test corrects for both the p-value and multiple comparisons, making it a conservative approach to prevent false positives. ANCOM-BC2 was performed with the sensitivity filter enabled to assess the effect of pseudo-counts on zeros. Pseudo-count methods are widely used to deal with the presence of zeros in data, however, the various approaches may influence the results [59]. Therefore, the sensitivity analysis determines if the statistical significance of the taxon was sensitive or not to the addition of psuedo-counts.

## Results

### Fish production and biometric data

The average weight of the fish at the beginning of the study (Time Point 1, T1) was 22.15 ± 0.24 grams (mean ± standard error [SEM]). At the six-week point in the growth trial, no significant differences in weight gain were observed between BSY1 (2% yeast) or BSY2 (0.75%) compared to the control, despite BSY2 having a 10% increase in weight gain over the control (Table 1). The production and biometric data for the BSF diets (Table 1) followed a similar trend to the BSY diets when compared to the control. No significant differences were found regarding weight gain, with BSF3 (old, 3%) having a 12% increase over the control and BSF4 (new, 0.75%) being 4% lower than the control. Production and biometric data for the mixed diet (BSY2 and BSF4 combination, 1.5%) also showed no significant differences regarding weight gain, with the mix having a 7% increase over the control (Table 1). Overall, no significant differences were found in the production and biometric parameters that were measured across the different fish treatment groups.

**Table 1:** Trout production (week 6) and biometric (week 7) data (individual level; mean ± standard error) of experimental treatment groups compared to the control.

Treatment	Survival Rate (%)	Weight Gain (g)	Weight difference vs. control (%)	FCR^†^
**Control**	100	31.8 ± 1.2	--	1.35 ± 0.03
**BSY1**	100	31.6 ± 1.0	−1%	1.35 ± 0.06
**BSY2**	98	35.0 ± 1.9	10%	1.35 ± 0.67
**Pooled Error**	--	2.83	--	0.11
**P-value**	--	0.21	--	0.99
**BSF3**	96	35.9 ± 2.8	12%	1.34 ± 0.67
**BSF4**	100	30.4 ± 1.4	−4%	1.35 ± 0.67
**Pooled Error**	--	3.86	--	0.19
**P-value**	--	0.19	--	0.99
**Mix**	92	34.1 ± 1.5	7%	1.32 ± 0.66
**Pooled Error**	--	2.71	--	0.08
**P-value**	--	0.27	--	0.56
**Treatment**	**Fillet Yields (%)**	**VSI** ^†^	**HSI** ^†^	**Fulton’s Condition Factor**
**Control**	54.8 ± 1.4	9.41 ± 0.33	1.78 ± 0.13	1.70 ± 0.06
**BSY1**	50.7 ± 1.4	9.40 ± 0.25	1.90 ± 0.13	1.64 ± 0.02
**BSY2**	54.4 ± 1.2	9.44 ± 0.30	2.08 ± 0.15	1.70 ± 0.05
**Pooled Error**	5.08	1.10	0.52	0.17
**P-value**	0.08	1.00	0.30	0.56
**BSF3**	55.6 ± 0.5	9.61 ± 0.22	2.01 ± 0.09	1.69 ± 0.05
**BSF4**	54.3 ± 1.2	9.33 ± 0.36	1.93 ± 0.09	1.65 ± 0.03
**Pooled Error**	4.21	1.15	0.39	0.18
**P-value**	0.71	0.81	0.30	0.75
**Mix**	52.8 ± 1.6	9.44 ± 0.33	1.78 ± 0.16	1.65 ± 0.04
**Pooled Error**	5.59	1.22	0.55	0.19
**P-value**	0.35	0.96	0.98	0.50

†Feed Conversion Ratio (FCR), Viscerosomatic Index (VSI) and Hepatosomatic Index (HSI), respectively.

### Stress and survival rates in relation to environmental conditions

The chiller used to maintain the water temperature of the RAS slowly began to decrease in functionality after a few weeks into the trial (S1 Fig). Additionally, the ambient temperature in the laboratory where the RAS was located (Blacksburg, VA, USA) lacked central air conditioning and water temperature began to increase (from 15 to 27˚C) as the weather changed seasons from early to late spring. The decrease in chiller effectiveness, compounded with an increase in ambient temperature, led to an unexpected rise in water temperatures (21.2 ± 0.3 ˚C) (associated with a decrease in dissolved oxygen) after week four (Table 2). This progressed for approximately two more weeks, with the average temperature rising to above the optimal growth range for rainbow trout [60]. Additional water quality parameters remained in a safe range throughout the duration of the trial and were as follows (average ± standard error of the mean [SEM]): 7.05 ± 0.07 pH, 0.42 ± 0.12 mg/L ammonia-N, 0.03 ± 0.01 mg/L nitrite-N, and 9.04 ± 1.06 mg/L nitrate-N [36,61].

**Table 2:** Trout production data (individual level; mean ± standard error) of all treatment groups compared to the control separated by two-week increments.

	Week 0-Week 2			Week 2-Week 4			Week 4-Week 6		
**Temperature** ^‡^	16.1 ± 0.4			18.6 ± 0.6			21.2 ± 0.3		
**DO** ^‡^	7.30 ± 0.16			6.64 ± 0.17			5.64 ± 0.08		
**FCR** ^§^	0.88 ± 0.03			1.02 ± 0.03			3.31 ± 0.73		
**Treatment**	**Survival (%)**	**Weight gain (g)**	**FCR** ^§^	**Survival (%)**	**Weight gain (g)**	**FCR** ^§^	**Survival (%)**	**Weight gain (g)**	**FCR** ^§^
**Control**	100	13.1 ± 0.9	0.75 ± 0.05	100	14.0 ± 0.9	1.05 ± 0.05	100	5.2 ± 1.1	4.51 ± 1.48
**BSY1**	100	10.8 ± 0.6	0.93 ± 0.05	100	14.1 ± 0.7	1.00 ± 0.04	100	7.2 ± 1.0	2.84 ± 0.51
**BSY2**	100	11.8 ± 1.2	0.87 ± 0.44	100	14.1 ± 1.4	1.05 ± 0.52	98	7.9 ± 0.9	2.72 ± 1.36
**BSF3**	100	12.1 ± 1.3	0.83 ± 0.42	100	14.0 ± 1.3	1.05 ± 0.53	96	9.2 ± 1.5	2.48 ± 1.24
**BSF4**	100	10.0 ± 1.3	0.99 ± 0.49	100	13.7 ± 1.2	0.97 ± 0.48	100	6.2 ± 1.6	4.96 ± 2.48
**Mix**	92	12.1 ± 0.6	0.89 ± 0.44	100	15.1 ± 0.9	0.97 ± 0.49	100	7.0 ± 1.3	3.26 ± 1.63
**Pooled error**	--	2.03	0.17	--	2.14	0.16	--	2.51	2.63
**P-value**	--	0.36	0.53	--	0.97	0.93	--	0.38	0.69

‡Temperature (˚C), Dissolved Oxygen (DO, mg/L), respectively.

§Feed Conversion Ratio (FCR).

The feed efficiency of the fish across all diets decreased over the course of the study as represented by the FCR, which increased drastically when the animals were under chronic stress. Despite repair maintenance on the chiller, the water temperatures continued to rise. The study was initially planned to be twelve weeks, but the timeline was cut short at week seven when the chiller ceased to function effectively due to a mechanical failure of the chiller’s freeze-thaw cycle, causing the coils to permanently freeze. Water temperatures and dissolved oxygen reached a lethal range (27˚C and 4 mg/L, respectively; [34] overnight, causing an acute stress event for the fish with high mortality rates. The total survival rate of all fish was 60%, but there were noticeable differences in the survival rate across the different diets (Fig 1).

**Fig 1:**
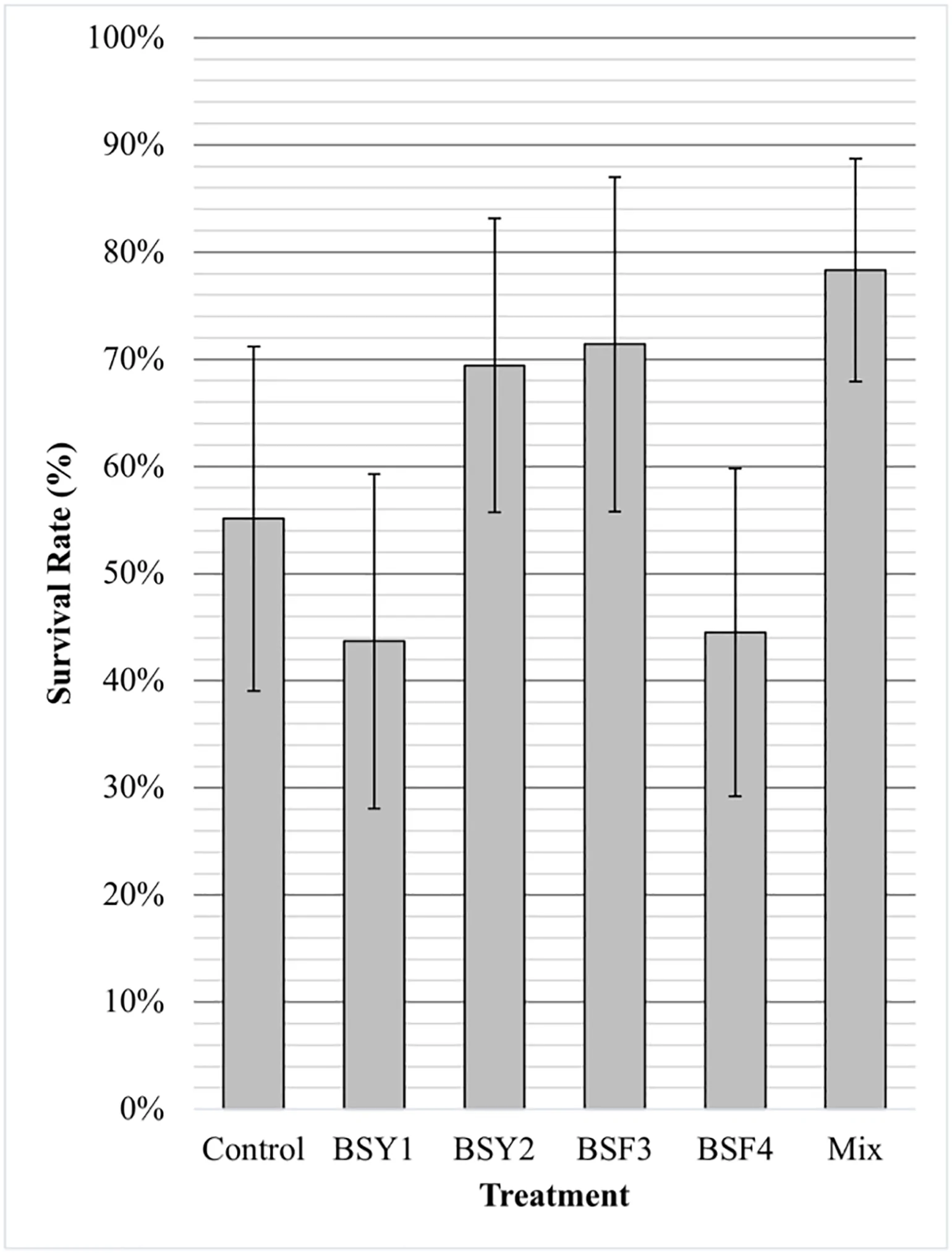
Acute stress event survival rate of all treatment groups. Average survival rates (%) were calculated based on the number of living fish the day prior to the overnight chiller failure, compared to the number of surviving fish the next day at the cessation of the trial. The number of surviving fish was recorded for each tank individually and then averaged according to diet. Error bars represent standard error of the mean (see the text for values).

Fish fed three of the five experimental diets showed higher survival rates on average when compared to the 55% survival level of the control group. The mixed diet had the highest survival rate at 78%, followed by BSF3 at 71% and BSY2 at 69%. The two experimental diets with lower survival rates than the control were BSF4 and BSY1 at 45% and 44%, respectively. Despite BSY2, BSF3, and the mixed diet having 14–23% higher survival rates on average compared to the control, no statistical significances (P > 0.05) were observed following an ANOVA test. There was a large amount of variance seen in the survival rates on a tank-by-tank basis for each diet. The SEM for average survival rate across all diets were as follows: 10% (Mix), 14% (BSY2), 15% (BSF4), and 16% (Control, BSY1, BSF3).

### Phylum-level relative abundance in the bacterial communities

The total number of sequences generated prior to filtering the 16S rRNA gene data was 7,231,114 reads. The resulting number of sequences after filtering was 5,354,452 reads, with 1,183 unique ASVs identified. The phylum-level relative abundance bar plot of the swabs from control fish was determined (Fig 2A). Fish fed the control diet were sampled at both T1 (Control(T1)) and T2. The sampling at T2 included fish that were euthanized (Control(T2)), and fish that underwent a natural death during the acute stress event just prior to the T2 harvest (Control(ND)). Control(T1) exhibited a very large dominance of Firmicutes, followed by a lower abundance of Spirochaetota. A major shift in dominant phyla is observed between Control(T1) and Control(T2), with Actinobacteriota and Proteobacteria becoming the dominant phyla at T2 and lower levels of the Firmicutes and Spirochaetota. Fusobacteriota became the overwhelmingly dominant phylum for the Control(ND) animals. The abundance of Proteobacteria noticeably increased in both T2 samples compared to T1. Additionally, Actinobacteriota decreased drastically between Control(T2) and Control(ND).

**Fig 2:**
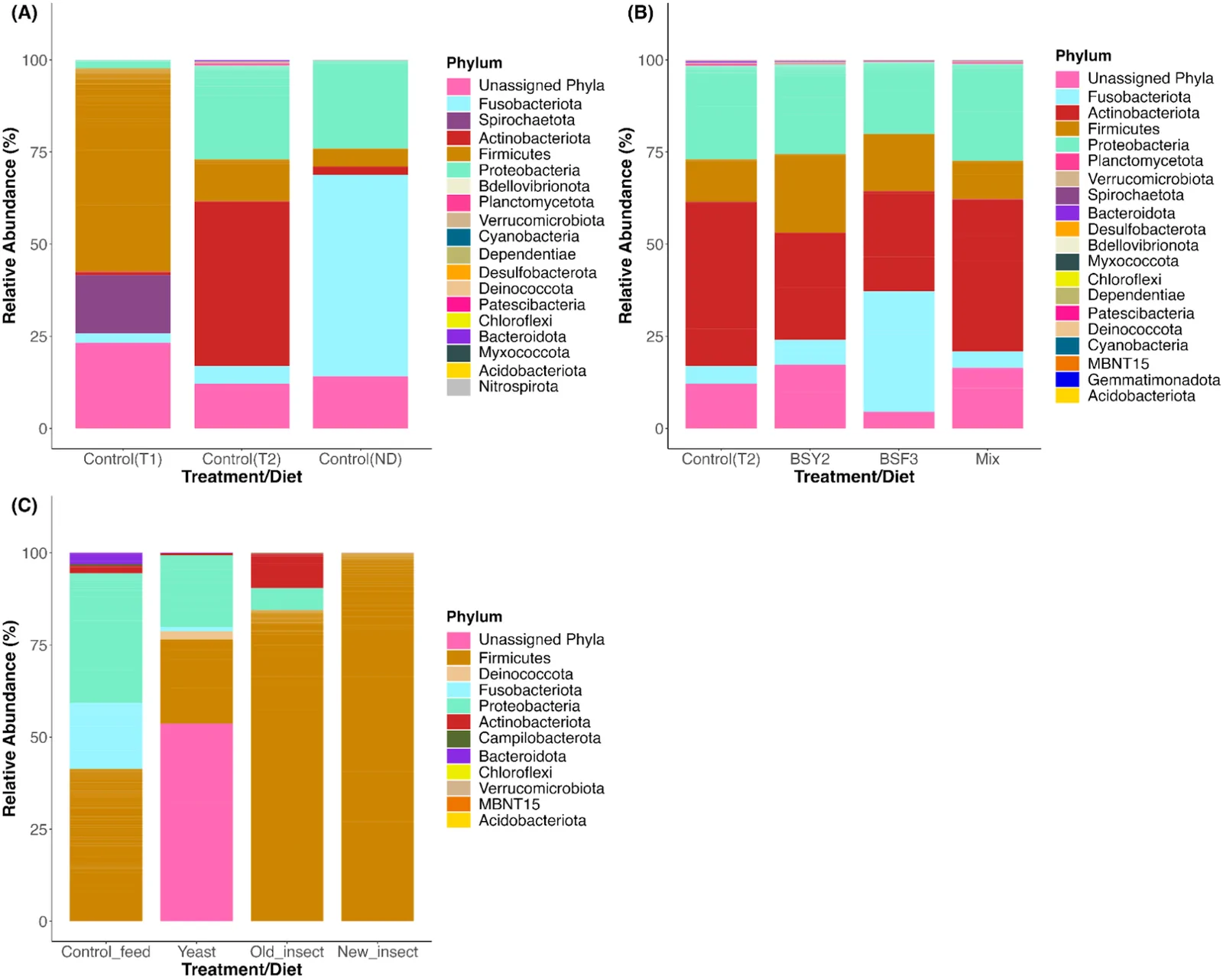
Phylum-level relative abundance bar plots of intestinal mucosa and feed bacterial communities. The 16S rRNA gene V4 region was sequenced from swab samples of the intestinal mucosa of rainbow trout, as well as from weighed samples of the control feed and feed additives. Mucosal samples from various time points of fish fed the control diet include: **(A)** time point 1 (Control(T1)), time point 2 euthanized (Control(T2)), and time point 2 natural death (Control(ND)). Intestinal samples of fish fed various diets taken at T2 include: **(B)** control, BSY2, BSF3, and mixed. Feed samples comprised of: **(C)** pellets of the control feed, the powdered yeast product, the “old” liquid insect product for BSF3, and the “new” liquid insect product for BSF4 and the mix. Bar plots were constructed using the average reads from the samples (n = 8 for all conditions except Control(T1), where n = 6, and the feed and feed additives, where n = 4) to illustrate relative diversity of the one hundred most abundant ASVs.

Next, the phylum-level relative abundance bar plot for fish with the highest survival rates from three of the experimental diets sampled at T2 was generated (Fig 2B). Like the Control(T2) results, the dominant phylum across all experimental diets was Actinobacteriota, followed by Proteobacteria. A high abundance of Fusobacteriota was seen in BSF3 relative to the other experimental diets. Overall, the Firmicutes were greatly diminished across all diets at T2 compared to what was observed in Control(T1).

Since the feed additives may have contributed to changes in the fish intestinal bacterial community, a phylum-level relative abundance bar plot of the control feed and feed additive ingredients was created to enable a comparative analysis (Fig 2C). The three feed additive samples consisted solely of the raw ingredients. Two dominant phyla were seen in the control feed pellets, Firmicutes and Proteobacteria. The powdered yeast product was mostly comprised of the unassigned phylum category, followed by a relatively equal abundance of Firmicutes and Proteobacteria. The old insect product (used for BSF3) and new insect product (used for BSF4 and the mix) were highly dominated by Firmicutes. Overall, there does not appear to be any strong correlation of the patterns between the dominant phyla of the raw feed additive ingredients and those of the intestinal mucosa samples from fish fed the associated diet.

Lastly, water column samples were taken to compare the external bacterial environment to the internal bacterial environment of the fish intestines (S2 Fig). The water column samples from T1 did not contain enough bacterial DNA to be processed or sequenced. Two dominant phyla were present in the T2 water samples, Proteobacteria and Bacteroidota. Proteobacteria appears to be the only similarity between the fish mucosa samples and the water column, as it was prevalent across all T2 mucosa samples (Fig 2B). Bar plots of each individual mucosa sample within the various diet groups illustrate the degree of variation observed between the bacterial microbiome of the individual fish (S3 Fig).

### Family-level relative abundance in the bacterial communities

A heatmap was made of the top 25 most relatively abundant ASVs, log-transformed at the family level (Fig 3). The samples taken at T1 consist of feces (Feces(T1)) and mucosal samples (Control(T1)) from the control diet-fed fish after the three-week acclimation period. A high abundance of *Streptococcaceae* and *Clostridiaceae* was seen in both samples. A lower, yet relatively equal, abundance of *Peptostreptococcaceae* and *Peptostreptococcales-Tissierellales* was seen in both feces and the mucosa as well. However, the mucosal samples had a higher abundance of *Brevinemataceae* and a lower abundance of *Fusobacteriaceae* and *Moraxellaceae* in comparison to the feces. Overall, the mucosal samples and the feces share some similar trends in their composition at the family level.

**Fig 3:**
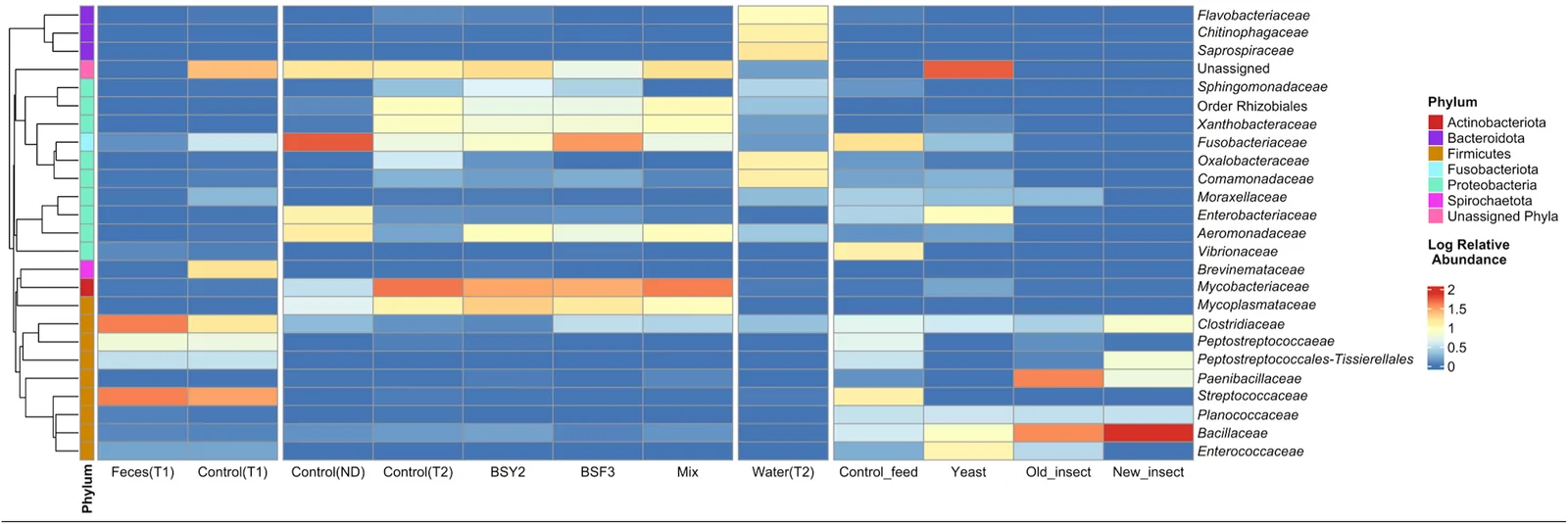
Family-level relative abundance heatmap of bacterial communities. The 16S rRNA gene V4 region was sequenced from various samples. Timepoint 1 includes: feces (Feces(T1); n = 4) and mucosa samples (Control(T1); n = 6) from fish fed the control diet. Timepoint 2 consists of: mucosa samples) from fish fed the control (natural death Control(ND); euthanized, Control(T2)), BSY2, BSF3, and mixed diets (n = 8 per condition), as well as the water column samples (Water(T2); n = 2). Lastly, the feed samples (n = 4 per diet) comprised of pellets of the control feed, the powdered yeast product, the “old” liquid insect product for BSF3, and the “new” liquid insect product for BSF4 and the mix. QIIME2 was used to construct the phylogenetic tree on the lefthand side of the figure. The log relative abundance of the top 25 family-level taxa is plotted and sorted by phylum using the phylogenetic tree.

The most abundant ASVs at the family level for T2 (Fig 3), are relatively consistent across all mucosal samples for the control and experimental euthanized fish. However, the T2 natural death fish (Control(ND)) noticeably differed from the euthanized fish in their intestinal bacterial communities. The two most dominant families across all the experimental diets were *Mycobacteriaceae* and *Mycoplasmataceae*. The most dominant family in Control(ND) was *Fusobacteriaceae*, which is seen across all other sample groups at a lower abundance, though higher levels in BSF3, comparatively. Furthermore, *Xanthobacteraceae* and the order Rhizobiales were also present at a higher relative abundance across the experimental groups, but essentially nonexistent in Control(ND). *Aeromonadaceae* was seen at higher levels in all T2 samples except Control(T2), in which it was noticeably lower.

Drastic shifts in the bacterial community between T1 and T2 were observed across almost every family where relative abundance was not equal to zero across all mucosal samples. The most abundant families in T1 were diminished at T2, and dominant T2 families such as *Mycobacteriaceae* were drastically lower or nonexistent at T1. The composition of the bacterial families present in the T1 versus the T2 mucosal samples is quite distinct.

Differential abundance analysis was performed on the control diet group using ANCOM-BC2 with Dunnett’s type of test to determine significant differences in taxa at the family level across timepoints. Both sample groups from T2 (Control(T2) and Control(ND)) were compared to the sample group from T1 (Control(T1); reference group), and the log fold-change (LFC) in abundance of taxa is presented in a heatmap (Fig 4). Two of the most abundant taxa at T1, *Streptococcaceae* and *Clostridiaceae*, both from the phylum Firmicutes, tremendously decreased in abundance at both T2 samples. Other prominent changes in organisms that passed the sensitivity test (regarding the addition of pseudo-counts on zero counts) include a significant increase of *Mycobacteriaceae* in Control(T2) and *Fusobacteriaceae*, *Enterobacteriaceae,* and *Aeromonadaceae* in Control(ND) that are highlighted in Fig 4. Various other taxa were identified as decreasing or increasing, too. Control(T2) and Control(ND) followed similar trends regarding increases and decreases in taxa when compared to Control(T1), though having some distinct differences between the degree of the LFC. ANCOM-BC2, using the global test function, was also performed on the experimental groups sampled at T2, but no significant differences in abundance were determined according to diet.

**Fig 4:**
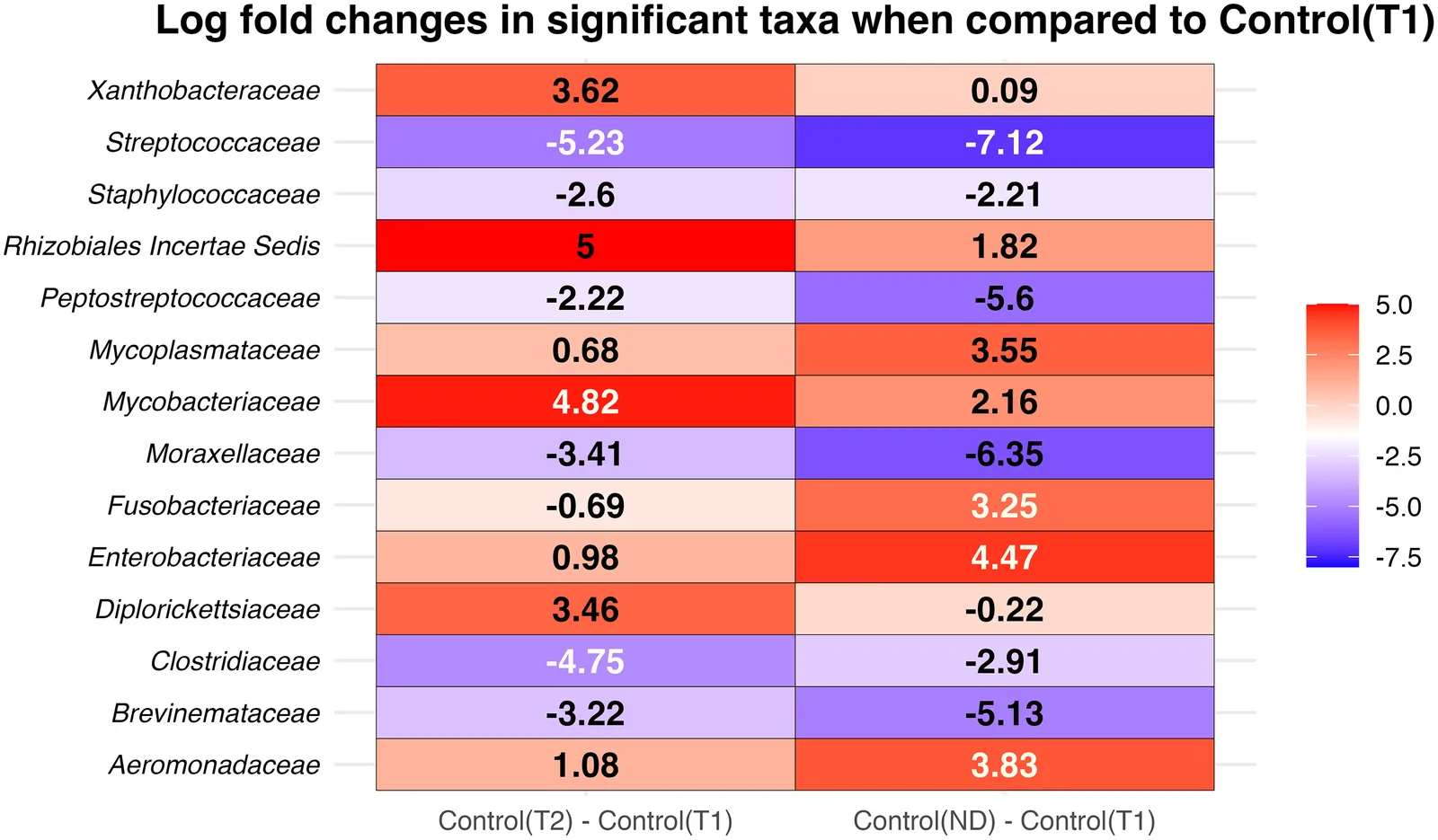
Heatmap of differential abundance showing significant taxonomic differences at the family level between intestinal bacterial communities of rainbow trout fed the control diet. Differential abundance was calculated via the analysis of compositions of microbiomes with bias correction 2 (ANCOM-BC2). The bacterial communities from the rainbow trout at T2 (euthanized, Control(T2), natural death, Control(ND); n = 8) were each compared to the bacterial community from the trout at T1 (Control(T1); n = 6) using Dunnett's type of test. The legend on the righthand side of the figure, as well as the value within each cell, represents the log fold-change (LFC) (in natural log) of family-level relative abundance in comparison to Control(T1). Higher LFC values indicate larger differences between the shift in abundance compared to the reference community. Positive values (red) mean higher abundance of the family in the context of the community shift, and vice versa for negative (blue). The values presented in white represent the taxa that passed the sensitivity test regarding the addition of pseudo-counts on zero counts during the analysis.

The water column sample from T2 had a considerably different bacterial composition when compared to the mucosal samples (Fig 3). Although highly abundant families from Proteobacteria in the mucosa samples do appear to be present in the water column, but at extremely low levels, and vice versa. Families from the phylum Bacteroidota were highly abundant in the water column, but not present in the mucosa, including *Flavobacteriaceae*, *Chitinophagaceae*, and *Saprospiraceae*. Overall, the water column had a very different bacterial composition at the family level when compared to the mucosal samples from T2, suggesting that it had little impact on the internal bacterial community of the fish.

The family-level relative abundance for each of the feed and feed additive samples had a distinctive pattern (Fig 3). The most abundant ASVs from the control feed pellets were in the families *Fusobacteriaceae*, *Vibrionaceae*, and *Streptococcaceae*. The powdered yeast product was comprised mostly of unassigned phylum members. The three most abundant assigned families were *Bacillaceae* (also high in both insect products), *Enterococcaceae*, and *Enterobacteriaceae*. Comparable levels between the yeast and insect products are also observed regarding the abundance of *Clostridiaceae* and *Planococcaceae*. However, the most dominant families for the old insect and new insect products were all from the phylum Firmicutes, with *Bacillaceae* being highly abundant in both. Between the two insect feed additives, *Paenibacillaceae* was higher in the old insect product, whereas the new insect product had higher levels of *Clostridiaceae* and *Peptostreptococcales-Tissierellales*. The insect products also differed regarding the presence of *Moraxellaceae* and *Enterococcaceae*, with lower abundance in the new insect product. A relatively equal abundance was seen for most other families. There is not any obvious relationship between the most abundant families in the raw feed additives and those abundant in the T2 intestinal mucosa samples from fish fed the associated diets.

### Alpha diversity analysis of the bacterial communities

Three alpha diversity analyses (Shannon, Evenness, and observed ASVs) were performed to measure the bacterial community diversity within each sample group (Table 3). For the Shannon index, higher values indicate higher diversity. Regarding evenness, the closer the value is to one, the more evenly distributed the community composition is. Observed ASVs just represents a count. Within the control diet group, there were not statistically significant (P > 0.05) differences. However, there was a decreasing trend in alpha diversity values from Control(T1) to Control(T2), with Control(ND) having the lowest value for each metric. The largest decline is seen in the number of observed ASVs, with Control(ND) having over a 70% decrease compared to Control(T1). Control(T2) had an almost 50% decrease when compared to Control(T1).

**Table 3:** Summary of alpha diversity analyses of intestinal mucosa samples (mean ± standard error).

	Diet/Treatment	Shannon	Evenness	Observed ASVs
**Control Diet**	**Control(T1)**	3.12 ± 1.73	0.52 ± 0.23	66.17 ± 45.84
	**Control(T2)**	2.52 ± 1.03	0.49 ± 0.15	36.75 ± 22.96
	**Control(ND)**	1.67 ± 0.74	0.43 ± 0.14	17.13 ± 13.63
	P-value^†^	0.143	0.290	0.062
**Experimental Diets**	**Control(T2)**	2.52 ± 1.03	0.49 ± 0.15	36.75 ± 22.96
	**BSY2**	2.41 ± 0.85	0.50 ± 0.14	28.43 ± 11.30
	**BSF3**	1.50 ± 0.75	0.35 ± 0.14	19.43 ± 10.28
	**Mix**	2.33 ± 0.27	0.51 ± 0.08	26.71 ± 10.77
	P-value^†^	0.069	0.042^‡^	0.252

†P-value of the one-way Kruskal-Wallis test.

‡No significant differences found after Dunn’s post-hoc test.

When comparing the experimental diets sampled at T2, BSF3 had the lowest diversity across all metrics. Control(T2) had the highest Shannon diversity, though relatively like BSY2, followed by the mixed diet. The same pattern is seen for observed ASVs, with Control(T2) having the most and BSY2 and the mixed diet having lower values. Evenness was approximately equal for all diets aside from BSF3, which was lower. No statistical significance (P > 0.05) was observed in any of the metrics. However, the evenness metric was initially significant following the one-way Kruskal-Wallis test (P < 0.05) prior to Dunn’s post-hoc test, but no significance was found after the pairwise comparisons due to the conservative nature of the Bonferroni correction, which aims to prevent false positives.

The alpha diversity of the feed and feed additive samples showed some statistically significant differences across various samples and metrics (S1 Table). Overall, differences were observed between the raw feed additives and the control feed pellets, but not between any of the raw feed additives themselves.

### Beta diversity analysis of the bacterial communities

Beta diversity analysis was performed between treatment groups using the UniFrac distances [62] to provide insight into the level of dissimilarity between bacterial communities,. Non-metric Multidimensional Scaling (NMDS) plots representing (1) unweighted and (2) weighted UniFrac distances were generated (Fig 5). Statistical differences were calculated using PERMANOVA, followed by a pairwise comparison with Bonferroni correction. For fish fed the control diet, both the unweighted (Fig 5A1) and weighted (Fig 5A2) NMDS plots help visualize that the bacterial community of Control(T1) is distinct (P < 0.01) when compared to both Control(T2) and Control(ND). In the unweighted NMDS plot, there is a large amount of overlap between Control(T2) and Control(ND), indicating comparable community compositions (P > 0.05). However, when using the weighted UniFrac distance which accounts for abundance, the two groups show a significant level (P < 0.01) of dissimilarity.

**Fig 5:**
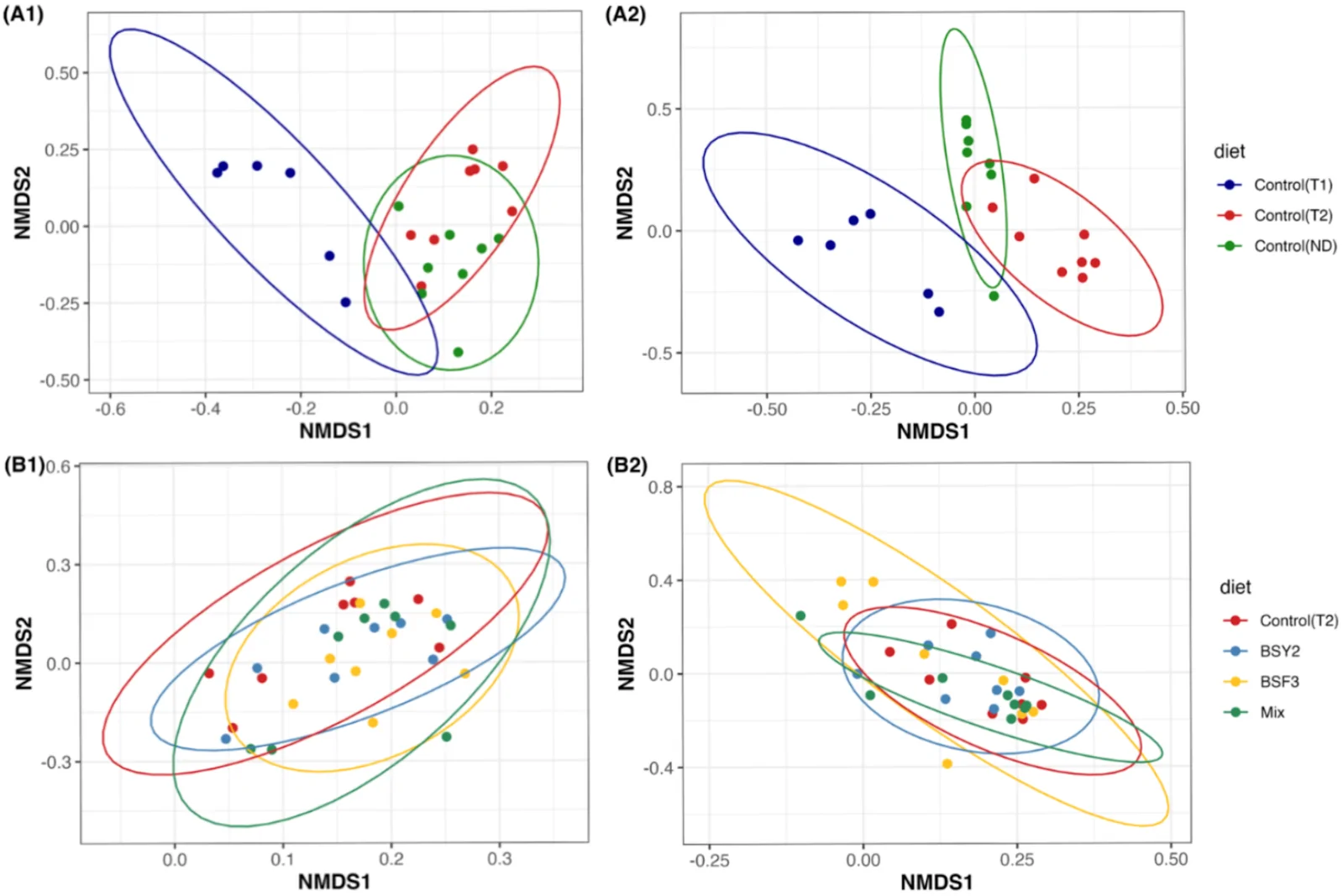
Non-metric Multidimensional Scaling (NMDS) plots representing unweighted and weighted beta diversities of intestinal bacterial communities. Dissimilarity was calculated using the **(1, left)** unweighted UniFrac and **(2**, **right)** weighted UniFrac distance. Intestinal mucosa samples from fish fed the control diet were sampled at: **(A)** time point 1 (Control(T1)), time point 2 euthanized (Control(T2)), and time point 2 natural death (Control(ND)). Intestinal mucosa samples of fish sampled at T2 are represented by the various diets as: **(B)** Control(T2), BSY2, BSF3, and mixed. Ellipses represent 95% confidence intervals that assume a multivariate t-distribution of eight intestinal mucosa samples of fish sampled from each condition, except for Control(T1), which consisted of six.

Diversity analysis of the T2 control and experimental diets shows a large amount of overlap in the unweighted NMDS plot between all four groups (Fig 5B1), demonstrating a large degree of likeness between the bacterial communities. The same is seen for the weighted UniFrac distance (Fig 5B2), though BSF3 appears to have larger range, comparatively. The PERMANOVA results revealed highly comparable bacterial communities (P = 1) for both the unweighted and weighted UniFrac distances across all treatments at T2.

For the feed and feed additive samples (S4 Fig), the initial PERMANOVA results indicated significant differences (P < 0.001), however, the pairwise comparison showed no significance between any of the groups when accounting for multiple testing. Though, prior to factoring in error correction accounting for multiple testing, the pairwise comparisons did show that the bacterial communities of the feed groups are all distinct (P < 0.05) amongst each other.

## Discussion

During this study, no significant differences in production data were observed between the euthanized fish fed the five experimental diets compared to the control. Although, when looking at the average weight gain and FCR during the first six weeks of the trial, three experimental diets (BSY2, BSF3 and MIX) performed better, on average, than the control (Tables 1–3) and the other two were comparable to the control. Biometric data (fillet yields, VSI, HSI, and Fulton’s Condition Factor) collected at week seven of the trial suggested no negative impacts of the feed additives on the physiology of the fish, but neither did the addition of BSY and BSF as feed additives significantly increase overall growth efficiency.

Previous research on the use of yeast and BSF in aquaculture feeds has often focused on using these protein sources as fishmeal replacements, as opposed to feed additives. Studies have found that high percentages (40%+) of yeast used as a fishmeal replacement in feed significantly decreased growth in species such as Atlantic salmon [63] and rainbow trout [17]. Conversely, when used in lower concentrations (≤ 20%), growth performance of rainbow trout increased or remained neutral [17,64]. When used as a feed additive, opposed to a fishmeal replacement, concentrations of yeast as low as 2–5% greatly increased growth in rainbow trout [65,66]. Results of BSF as a fishmeal replacement in feed for salmonids vary greatly, with studies reporting increased, comparable, or decreased growth performance; though the majority of studies found no negative health effects [11,13,14,67]. Overall, the use of BSY or BSF as solely feed additives, as opposed to partial or complete fishmeal replacements, is much less common.

The results of the current trial were negatively impacted by the increase in water temperature and associated decrease in dissolved oxygen, which were unplanned chronic stress variables that arose after week four of the trial. Additionally, the trial was ended five weeks early because of the acute stress event, limiting the amount of data that was obtained. Though the stress factors were not controlled for, all fish were subjected to the same conditions throughout the duration of the study due to the nature of the RAS. Notable trends in per-diet feed efficiency appear when looking at the data separated into biweekly increments (Table 2). In the first two weeks under optimum water quality (Week 0-Week 2), the control fish gained the most weight, which may be attributed to not having to acclimate to a new feed. However, the following two weeks (Week 2-Week 4), all diets gained approximately equal weight, and some of the experimental diets exhibited better FCR values than the control. Most importantly, during the last two weeks (Week 4-Week 6) under chronic thermal stress, all the experimental diets outperformed the control diet (though not statistically significant, P > 0.05) regarding both weight gain and FCR, apart from the FCR for BSF4 which was higher. It is hypothesized that the higher temperature heat treatment used during the production of BSF4 may have damaged the quality of the feed additive, which is a trend that has been observed by the producer of the product, Maltento. If the duration of the trial had not been cut short, and the trends observed in Table 2 remained, the fish fed the experimental diets may have continued to further surpass the growth of the control fish, in turn leading to significant differences in production efficiency. Overall, the results indicate that the fish were better able to utilize the feed coated with feed additives while experiencing both chronic and acute stress, when compared to the control.

When rainbow trout are subjected to thermal stress, a physiological stress response is induced in the fish that is associated with reduced food intake, growth inhibition, and severe damage to the intestinal tract [68]. High-temperature stress is also known to cause metabolic disorders and decreased immune function in rainbow trout, negatively impacting fish physiology and ultimately leading to death in extreme temperatures [69,70]. Regarding stress-related mortality, three of the experimental diets (BSY2, BSF3, Mix) exhibited higher survival rates (over 14–23% greater) compared to the control during the acute stress event, although not statistically significant (P > 0.05; Fig 1). The same three diets with the highest survival rate from the acute stress event also gained the most weight after six weeks of the trial. The results from this study indicate that the feed additives (both BSY and BSF) may have had a positive impact on feed efficiency and production of rainbow trout when under stress conditions, possibly by increasing the health and robustness of the fish via immunoregulation [29,32] or increased feed palatability, leading to higher feed intake [71].

The bacterial composition of the feed additives, largely dominated by Firmicutes (particularly *Bacillaceae*), did not appear to correlate with the changes in the intestinal bacterial community observed in the fish at T2, nor did the water column (Figs 2 and 3; S2 Fig). Highly abundant families from the water column, within the phylum Bacteroidota and Proteobacteria, have been found as common isolates from water sources [72,73].

Beta diversity analysis revealed that the intestinal mucosa-associated bacterial communities of the rainbow trout were vastly different between time points T1 and T2 (P < 0.01), but not between diets (P > 0.5). The observed timepoint differences likely reflect the effects of thermal stress (Fig 5). Significant differences (P < 0.01) were also found in the weighted diversity analysis between Control(T2) euthanized versus Control(ND) natural death fish, which accounts for bacterial species abundance on top of just presence/absence. Alpha diversity analysis, which looks at bacterial diversity within sample groups, found no significant differences across time points or diets (Table 3). However, a trend was seen in the control diet group in which a decline of all alpha diversity metrics occurred over time and as animal health declined. This agrees with previous research where less diversity, richness, and number of taxa in rainbow trout were observed under thermal stress conditions compared to normal rearing temperatures [68,74].

Interestingly, although BSF3 was found to have the lowest alpha diversity metrics across all the experimental diets, it exhibited the highest weight gain of the experimental diets both during chronic stress (Week 4-Week 6) and total (Week 0-Week 6). Although lower diversity typically has negative connotations, the results may indicate that high levels of a possibly beneficial microorganism could have led to out-competition of various other potentially harmful microorganisms, reducing the overall bacterial diversity. The ASV table (S2 Table) revealed that BSF3 had a very high abundance of *Cetobacterium* sp., which was four to five-fold higher than the other T2 diets. Further, when compared to Control(T2), BSF3 had a 39% reduction in *Mycobacterium* sp., a potential pathogen discussed in detail below. Although commonly found in high abundance, the role of *Cetobacterium* within the intestinal tract of rainbow trout needs further research. One study exploring the effects of diet supplementation and handling-stress in rainbow trout suggested *Cetobacterium* may be important for the metabolism of certain nutrients and vitamins, but also discussed the necessity for further investigations of the exact role this organism plays within the intestinal tract [75].

At the phylum level, all diets sampled at timepoint T2 appeared to be quite consistent (Fig 2B), apart from BSF3 having a higher abundance of Fusobacteriota compared to the rest. However, clear shifts in the dominant bacterial phyla between timepoint controls (Control(T1) vs. Control(T2)/(ND)) were observed (Fig 2A), with evident changes being increasing abundances of Actinobacteriota and Proteobacteria, and the diminishing of Firmicutes at Control(T2). Firmicutes have commonly been found to be a core constituent of the intestinal bacterial community in various fish species, encompassing diverse families including both beneficial lactic acid bacteria such as *Lactococcus lactis*, as well as fish-specific pathogens [66,76–79]. Proteobacteria is also very commonly reported across the literature as being a large component of the fish gastrointestinal tract, and contains diverse members that can be both beneficial or pathogenic [79]. When looking at Control(ND), the natural death fish, the same decrease in Firmicutes and increase in Proteobacteria as Control(T2) was seen compared to Control(T1), but the dominant phyla became Fusobacteriota. For both BSF3 and Control(ND), the organism *Cetobacterium* sp. constituted essentially the entirety of the Fusobacteriota phylum (S2 Table). As described above, higher *Cetobacterium* sp. levels typically correlate with increased fish health [75,80,81]. Therefore, the reason for the high abundance of *Cetobacterium* sp. in Control(ND) is unknown, especially because it appeared to be a possibly beneficial organism in the BSF3 diet. However, the presence of potential pathogens such as *Aeromonas* sp., which were notably higher (3-fold) in Control(ND) when compared to BSF3 likely played a role.

The family-level heatmap (Fig 3) and differential abundance analysis (Fig 4) revealed specific changes between time point controls (Control(T1) and Control(T2)) within the phylum Firmicutes, including families such as *Clostridiaceae*, *Streptococcaceae*, and *Mycoplasmataceae*. Interestingly, *Mycoplasma* sp. (family *Mycoplasmataceae*), very commonly seen and classified as a commensal organism in the fish intestinal tract [79], greatly increased in relative abundance in both Control(T2) and Control(ND) compared to Control(T1) (Figs 3 and 4, S2 Table). Increased abundance of *Mycoplasma* in stressed fish has been observed in prior research concerning thermal stress and the rainbow trout microbiome; though, conflicting results have been seen regarding high abundances of *Mycoplasma* correlating to both healthy and unhealthy fish [68,74]. The reasons are unknown. *Clostridiaceae* and *Streptococcaceae* significantly decreased in Control(T2) and Control(ND); both families include pathogenic and beneficial organisms. Minor decreases in many organisms from the family *Streptococcaceae* occurred, which collectively created the large family-level shift. In contrast, the shift in *Clostridiaceae* appeared to consist of the decrease of one organism, *Clostridium sensu stricto* 1 sp. (S2 Table). Similarly, the significant increase in the family *Fusobacteriaceae* (Phylum Fusobacteriota, discussed above) in Control(ND) was also attributed to changes in only one organism as well, *Cetobacterium* sp.. *Enterobacteriaceae*, which encompasses many foodborne and zoonotic pathogens, significantly increased in Control(ND) [82]. Identification past the family level was not assigned by the taxonomic classifier, therefore it is unknown whether the shift occurred due to one organism or a collection of many.

The wide variety of family level shifts, many of which were not discussed, helps demonstrate the extreme dysbiosis that occurred after the fish were subjected to high water temperatures for extended periods of time. Additionally, the impact that the abundance of one organism can have on a community is exemplified by some significant shifts. On the flip side, many organisms within the same family can be simultaneously affected as well, likely due to similar growth characteristics and requirements. Major shifts in the intestinal community likely allowed for the proliferation of opportunistic organisms that would typically be suppressed or outcompeted by the beneficial commensals. It is known that intestinal dysbiosis is associated with disease in humans and animals, with the reduction of beneficial organisms allowing for the proliferation of pathogenic species [83]. An emergence of potential pathogens *Mycobacteriaceae* and *Aeromonadaceae* was observed during this study.

Differential abundance analysis confirmed statistically significant increases in the abundance of *Mycobacteriaceae* between Control(T2) compared to Control(T1) (Fig 4). The ASV table of the most abundant organisms revealed that *Mycobacterium* sp. (family *Mycobacteriaceae*) represented virtually the entirety of Actinobacteriota observed in the T2 diets (Fig 2B), with the remaining organisms within that phylum having negligible relative abundances (S2 Table). A large range of *Mycobacterium* species are associated with pertinent disease outbreaks in many animals including fish and humans, with the most common aquatic bacterial species being the zoonotic *Mycobacterium marinum* [84]. Mycobacterial infections (mycobacteriosis) are a major concern in aquaculture and cause mass-mortality associated with large economic losses, as *Mycobacterium* species are extremely challenging to eradicate and control [85–87]. While the emergence and high abundance of *Mycobacterium* sp. in all sampled T2 fish is likely attributed to intestinal dysbiosis caused by chronic stress [88,89], the etiology cannot be proven in this study. Vaccination against mycobacterial infections in fish are uncommon due to ineffectiveness, and treatment using antibiotics is difficult due to the nature of *Mycobacterium* spp. compounded with the increase of antibiotic resistance; prophylactic treatment and prevention is a viable alternative mitigation strategy [84].

A significant increase in *Aeromonadaceae* occurred as well between the Control(ND) fish compared to Control(T1) and was also observed in fish fed the experimental diets sampled at T2. *Aeromonas* spp. encompasses various organisms that are pathogenic to both fish and humans, such as the prominent aquaculture pathogen of major concern, *Aeromonas hydrophila*, in which antibiotics are no longer effective at controlling [90,91]. Development of alternative treatments to antibiotics such as prebiotics and probiotics offer promise.

## Conclusion

In conclusion, using BSY and BSF cultured on BSG as feed additives in the diets of rainbow trout did not significantly affect production efficiency. However, an unplanned thermal stress became a major factor during the trial, occurring at both a chronic (two-week) and acute (12–24 hr) level. Significant differences in the intestinal mucosa-associated bacterial community were observed between the time point T1 and T2 controls, but not between any of the diets sampled at time point T2. The emergence of the potential pathogen *Mycobacterium* sp. in the bacterial communities of all time point T2 fish was likely a result of stress-induced intestinal dysbiosis. The bacterial communities of the feed additives did not appear to correlate with the shifts in the fish intestinal bacterial communities at time point T2, nor did the composition of the water column. Increased growth and survival rates were observed in three of the experimental diets (BSY2, BSF3, and mix) when under stress, indicating that the feed additives may have increased the health and robustness of the fish when subjected to these conditions. Further research is needed in a controlled stress trial and/or a pathogen challenge experiment to evaluate if the observed benefits can be attributed to possible prebiotic effects of the feed additives.

## Supporting information

S1 Fig Water temperature (˚C) throughout the duration of the trial (days). The colored circles represent different time points in the trial, as follows: blue, Time point 1 (T1); green, Week 2; yellow, Week 4; orange, Week 6; and red, Time point 2 (T2). The chronic stress is seen between the orange and red circles (Week 4 to 6), with the acute stress event and end of the trial being represented by the red circle.
(TIF)

S2 Fig Phylum-level relative abundance bar plot of bacterial communities. The 16S rRNA gene V4 region was sequenced from swab samples of the intestinal mucosa of rainbow trout, as well as from the retentate of the water column produced after vacuum filtration. The bacterial community of the mucosal samples from fish fed the control diet sampled at T2 (Control(T2); n = 8) are compared next to the water column sampled at T2 (Water(T2); n = 2). Bars were constructed using the average reads from the samples to illustrate relative diversity of the one hundred most abundant ASVs.
(TIFF)

S3 Fig Phylum-level relative abundance bar plots of individual sample bacterial communities. The 16S rRNA gene V4 region was sequenced from swab samples of the intestinal mucosa of rainbow trout. Individual mucosa samples within each diet group (n = 8 except Control(T1), where n = 6) are presented as follows: **(A)** Control(T1), **(B)** Control(T2), **(C)** Control(ND), **(D)** BSY2, **(E)** BSF3, and **(F)** Mix. Bars were constructed using the reads from the individual samples to illustrate relative diversity of the one hundred most abundant ASVs.
(TIFF)

S4 Fig Non-metric Multidimensional Scaling (NMDS) plots representing unweighted and weighted beta diversities of feed bacterial communities. Dissimilarity was calculated using the **(top)** unweighted UniFrac and **(bottom)** weighted UniFrac distance. Feed samples (n = 4 per diet) comprised of: **(C)** pellets of the control feed, the “new” liquid insect product used for BSF4, the “old” liquid old insect product used for BSF3, and the powdered yeast product. Ellipses represent 95% confidence intervals that assume a multivariate t-distribution of four samples of each feed additive.
(TIFF)

S1 Table Summary of alpha diversity analyses of feed (mean ± standard error). (DOCX)

S2 Table Amplicon sequence variant relative abundance table (mean ± SEM) of trout intestinal samples ordered by the overall highest average read count of organisms based on Control(T2). (DOCX)

## References

[1] Khalili Tilami S, Sampels S. Nutritional value of fish: Lipids, proteins, vitamins, and minerals. Rev Fish Sci Aquacult. 2017;26(2):243–53. doi: 10.1080/23308249.2017.1399104

[2] FAO. The State of World Fisheries and Aquaculture 2022: Towards Blue Transformation. Rome, Italy: FAO; 2022. doi: 10.4060/cc0461en

[3] D’Agaro E, Gibertoni P, Esposito S. Recent trends and economic aspects in the rainbow trout (Oncorhynchus mykiss) sector. Appl Sci. 2022;12(17):8773. doi: 10.3390/app12178773

[4] FAO. Fisheries and Aquaculture - Cultured Aquatic Species - Oncorhynchus mykiss. In: Food and Agriculture Organization of the United Nations [Internet]. 2023 [cited 3 Apr 2023]. Available: https://www.fao.org/fishery/en/culturedspecies/oncorhynchus_mykiss/en

[5] Verdile N, Pasquariello R, Scolari M, Scirè G, Brevini TAL, Gandolfi F. A detailed study of rainbow trout (Onchorhynchus mykiss) intestine revealed that digestive and absorptive functions are not linearly distributed along its length. Animals (Basel). 2020;10(4):745. doi: 10.3390/ani10040745 32344584 PMC7223369

[6] FAO. Blue Transformation - Roadmap 2022–2030: A Vision for FAO’s Work on Aquatic Food Systems. Rome, Italy: FAO; 2022. doi: 10.4060/cc0459en

[7] Olajire AA. The brewing industry and environmental challenges. J Clean Prod. 2020;256:102817. doi: 10.1016/j.jclepro.2012.03.003

[8] Rachwał K, Waśko A, Gustaw K, Polak-Berecka M. Utilization of brewery wastes in food industry. PeerJ. 2020;8:e9427. doi: 10.7717/peerj.9427 32742775 PMC7367049

[9] Kerby C, Vriesekoop F. An overview of the utilisation of brewery by-products as generated by british craft breweries. Beverages. 2017;3(2):24. doi: 10.3390/beverages3020024

[10] Mussatto SI, Dragone G, Roberto IC. Brewers’ spent grain: generation, characteristics and potential applications. J Cereal Sci. 2006;43(1):1–14. doi: 10.1016/j.jcs.2005.06.001

[11] St‐Hilaire S, Sheppard C, Tomberlin JK, Irving S, Newton L, McGuire MA, et al. Fly Prepupae as a Feedstuff for Rainbow Trout, Oncorhynchus mykiss. J World Aquaculture Soc. 2007;38(1):59–67. doi: 10.1111/j.1749-7345.2006.00073.x

[12] Makkar HPS, Tran G, Heuzé V, Ankers P. State-of-the-art on use of insects as animal feed. Ani Feed Sci Technol. 2014;197:1–33. doi: 10.1016/j.anifeedsci.2014.07.008

[13] Henry M, Gasco L, Piccolo G, Fountoulaki E. Review on the use of insects in the diet of farmed fish: Past and future. Anim Feed Sci Technol. 2015;203:1–22. doi: 10.1016/j.anifeedsci.2015.03.001

[14] Belghit I, Liland NS, Gjesdal P, Biancarosa I, Menchetti E, Li Y, et al. Black soldier fly larvae meal can replace fish meal in diets of sea-water phase Atlantic salmon (Salmo salar). Aquaculture. 2019;503:609–19. doi: 10.1016/j.aquaculture.2018.12.032

[15] Jucker C, Leonardi MG, Rigamonti I, Lupi D, Savoldelli S. Brewery’s waste streams as a valuable substrate for Black Soldier Fly Hermetia illucens (Diptera: Stratiomyidae). J Entomol Acarol Res. 2020;51(3). doi: 10.4081/jear.2019.8876

[16] Øverland M, Skrede A. Yeast derived from lignocellulosic biomass as a sustainable feed resource for use in aquaculture. J Sci Food Agric. 2017;97(3):733–42. doi: 10.1002/jsfa.8007 27558451

[17] Hines IS, Ferguson CS, Bushman TJ, Gatlin DM III, Jensen RV, Smith SA, et al. Impact of a yeast‐based dietary supplement on the intestinal microbiome of rainbow trout,Oncorhynchus mykiss. Aquacul Res. 2020;52(4):1594–604. doi: 10.1111/are.15011

[18] Kaushik S, Troell M. Taking the fish-in fish-out ratio a step further. Aquacult Eur. 2010;35:15–7.

[19] Marson GV, de Castro RJS, Belleville M-P, Hubinger MD. Spent brewer’s yeast as a source of high added value molecules: A systematic review on its characteristics, processing and potential applications. World J Microbiol Biotechnol. 2020;36(7):95. doi: 10.1007/s11274-020-02866-7 32583032

[20] Hamid R, Khan MA, Ahmad M, Ahmad MM, Abdin MZ, Musarrat J, et al. Chitinases: An update. J Pharm Bioallied Sci. 2013;5(1):21–9. doi: 10.4103/0975-7406.106559 23559820 PMC3612335

[21] Brandt KK, Amézquita A, Backhaus T, Boxall A, Coors A, Heberer T, et al. Ecotoxicological assessment of antibiotics: A call for improved consideration of microorganisms. Environ Int. 2015;85:189–205. doi: 10.1016/j.envint.2015.09.013 26411644

[22] Ringø E, Myklebust R, Mayhew TM, Olsen RE. Bacterial translocation and pathogenesis in the digestive tract of larvae and fry. Aquaculture. 2007;268(1–4):251–64. doi: 10.1016/j.aquaculture.2007.04.047

[23] Xiong J-B, Nie L, Chen J. Current understanding on the roles of gut microbiota in fish disease and immunity. Zool Res. 2019;40(2):70–6. doi: 10.24272/j.issn.2095-8137.2018.069 29976843 PMC6378566

[24] Tang Q, Jin G, Wang G, Liu T, Liu X, Wang B, et al. Current sampling methods for gut microbiota: A call for more precise devices. Front Cell Infect Microbiol. 2020;10:151. doi: 10.3389/fcimb.2020.00151 32328469 PMC7161087

[25] He S, Wang Q, Li S, Ran C, Guo X, Zhang Z, et al. Antibiotic growth promoter olaquindox increases pathogen susceptibility in fish by inducing gut microbiota dysbiosis. Sci China Life Sci. 2017;60(11):1260–70. doi: 10.1007/s11427-016-9072-6 28674769

[26] Lyons PP, Turnbull JF, Dawson KA, Crumlish M. Phylogenetic and functional characterization of the distal intestinal microbiome of rainbow trout Oncorhynchus mykiss from both farm and aquarium settings. J Appl Microbiol. 2017;122(2):347–63. doi: 10.1111/jam.13347 27860093

[27] Davani-Davari D, Negahdaripour M, Karimzadeh I, Seifan M, Mohkam M, Masoumi SJ, et al. Prebiotics: Definition, types, sources, mechanisms, and clinical applications. Foods. 2019;8(3):92. doi: 10.3390/foods8030092 30857316 PMC6463098

[28] Hoseinifar SH, Esteban MÁ, Cuesta A, Sun Y-Z. Prebiotics and Fish Immune Response: A Review of Current Knowledge and Future Perspectives. Rev Fisheries Sci Aquacult. 2015;23(4):315–28. doi: 10.1080/23308249.2015.1052365

[29] Staykov Y, Spring P, Denev S, Sweetman J. Effect of a mannan oligosaccharide on the growth performance and immune status of rainbow trout (Oncorhynchus mykiss). Aquacult Int. 2007;15:153–61. doi: 10.1007/s10499-007-9096-z

[30] Iliev DB, Liarte CQ, MacKenzie S, Goetz FW. Activation of rainbow trout (Oncorhynchus mykiss) mononuclear phagocytes by different pathogen associated molecular pattern (PAMP) bearing agents. Mol Immunol. 2005;42(10):1215–23. doi: 10.1016/j.molimm.2004.11.023 15829310

[31] Løvoll M, Fischer U, Mathisen GS, Bøgwald J, Ototake M, Dalmo RA. The C3 subtypes are differentially regulated after immunostimulation in rainbow trout, but head kidney macrophages do not contribute to C3 transcription. Vet Immunol Immunopathol. 2007;117(3–4):284–95. doi: 10.1016/j.vetimm.2007.03.005 17449114

[32] Fierro-Castro C, Barrioluengo L, López-Fierro P, Razquin BE, Villena AJ. Fish cell cultures as in vitro models of inflammatory responses elicited by immunostimulants. Expression of regulatory genes of the innate immune response. Fish Shellfish Immunol. 2013;35(3):979–87. doi: 10.1016/j.fsi.2013.07.015 23872473

[33] Tukmechi A, Bandboni M. Effects of Saccharomyces cerevisiae supplementation on immune response, hematological parameters, body composition and disease resistance in rainbow trout, Oncorhynchus mykiss (Walbaum, 1792). J Appl Ichthyol. 2013;30(1):55–61. doi: 10.1111/jai.12314

[34] Myrick CA, Cech JJ Jr. Temperature influences on California rainbow trout physiological performance. Fish Physiol Biochem. 2000;22(3):245–54. doi: 10.1023/a:1007805322097

[35] Leary S, Pharmaceuticals F, Underwood W, Anthony R, Cartner S, Johnson CL, et al. AVMA Guidelines for the Euthanasia of Animals: 2020 Edition. 2020.

[36] Bridgewater L, Association APH, Association AWW, Federation WE. Standard Methods for the Examination of Water and Wastewater. American Public Health Association; 2012.

[37] Pough II JD, Hines IS, Layton AR, Smith SA, Huang H, O’Keefe SF. Novel application of brewer’s spent byproduct serve as an effective feed additive to rainbow trout Oncorhynchus mykiss. Paper presented at: Aquaculture 2025; March 7, 2025. New Orleans, LA: 2025.

[38] Caporaso JG, Lauber CL, Walters WA, Berg-Lyons D, Lozupone CA, Turnbaugh PJ, et al. Global patterns of 16S rRNA diversity at a depth of millions of sequences per sample. Proc Natl Acad Sci U S A. 2011;108 Suppl 1(Suppl 1):4516–22. doi: 10.1073/pnas.1000080107 20534432 PMC3063599

[39] Hines IS, Markov Madanick J, Smith SA, Kuhn DD, Stevens AM. Analysis of the core bacterial community associated with consumer-ready Eastern oysters (Crassostrea virginica). PLoS One. 2023;18(2):e0281747. doi: 10.1371/journal.pone.0281747 36812164 PMC9946220

[40] Bolyen E, Rideout JR, Dillon MR, Bokulich NA, Abnet CC, Al-Ghalith GA, et al. Reproducible, interactive, scalable and extensible microbiome data science using QIIME 2. Nat Biotechnol. 2019;37(8):852–7. doi: 10.1038/s41587-019-0209-9 31341288 PMC7015180

[41] Callahan BJ, McMurdie PJ, Rosen MJ, Han AW, Johnson AJA, Holmes SP. DADA2: High-resolution sample inference from Illumina amplicon data. Nat Methods. 2016;13(7):581–3. doi: 10.1038/nmeth.3869 27214047 PMC4927377

[42] Quast C, Pruesse E, Yilmaz P, Gerken J, Schweer T, Yarza P, et al. The SILVA ribosomal RNA gene database project: improved data processing and web-based tools. Nucleic Acids Res. 2013;41:D590–6. doi: 10.1093/nar/gks1219 23193283 PMC3531112

[43] Yilmaz P, Parfrey LW, Yarza P, Gerken J, Pruesse E, Quast C, et al. The SILVA and “All-species Living Tree Project (LTP)” taxonomic frameworks. Nucleic Acids Res. 2014;42:D643–8. doi: 10.1093/nar/gkt1209 24293649 PMC3965112

[44] Glöckner FO, Yilmaz P, Quast C, Gerken J, Beccati A, Ciuprina A, et al. 25 years of serving the community with ribosomal RNA gene reference databases and tools. J Biotechnol. 2017;261:169–76. doi: 10.1016/j.jbiotec.2017.06.1198 28648396

[45] R Core Team. R: A Language and Environment for Statistical Computing. Vienna, Austria: R Foundation for Statistical Computing; 2023.

[46] Bisanz JE. qiime2R: Importing QIIME2 artifacts and associated data into R sessions. 2018. Available: https://github.com/jbisanz/qiime2R

[47] McMurdie PJ, Holmes S. phyloseq: An R package for reproducible interactive analysis and graphics of microbiome census data. PLoS One. 2013;8(4):e61217. doi: 10.1371/journal.pone.0061217 23630581 PMC3632530

[48] Oksanen J, Simpson GL, Blanchet FG, Kindt R, Legendre P, Minchin PR, et al. vegan: Community Ecology Package. 2022. Available from: https://cran.r-project.org/web/packages/vegan/index.html

[49] Wickham H. ggplot2: Elegant Graphics for Data Analysis. New York: Springer-Verlag; 2016.

[50] Gu Z, Eils R, Schlesner M. Complex heatmaps reveal patterns and correlations in multidimensional genomic data. Bioinformatics. 2016;32(18):2847–9. doi: 10.1093/bioinformatics/btw313 27207943

[51] Wickham H, François R, Henry L, Müller K, Vaughan D, Software P, et al. dplyr: A Grammar of Data Manipulation. 2023. Available from: https://cloud.r-project.org/web/packages/dplyr/index.html

[52] Pedro MA. pairwiseAdonis: Pairwise Multilevel Comparison using Adonis. 2020. Available: https://github.com/pmartinezarbizu/pairwiseAdonis

[53] Lin H, Peddada SD. Multigroup analysis of compositions of microbiomes with covariate adjustments and repeated measures. Nat Methods. 2024;21(1):83–91. doi: 10.1038/s41592-023-02092-7 38158428 PMC10776411

[54] Lin H, Peddada SD. Analysis of compositions of microbiomes with bias correction. Nat Commun. 2020;11(1):3514. doi: 10.1038/s41467-020-17041-7 32665548 PMC7360769

[55] Lin H, Eggesbø M, Peddada SD. Linear and nonlinear correlation estimators unveil undescribed taxa interactions in microbiome data. Nat Commun. 2022;13(1):4946. doi: 10.1038/s41467-022-32243-x 35999204 PMC9399263

[56] Dunnett CW. A multiple comparison procedure for comparing several treatments with a control. J Am Stat Assoc. 1955;50(272):1096–121. doi: 10.1080/01621459.1955.10501294

[57] Dunnett CW, Tamhane AC. Step-down multiple tests for comparing treatments with a control in unbalanced one-way layouts. Stat Med. 1991;10(6):939–47. doi: 10.1002/sim.4780100614 1876783

[58] Dunnett CW, Tamhane AC. A step-up multiple test procedure. J Am Stat Assoc. 1992;87(417):162–70. doi: 10.1080/01621459.1992.10475188

[59] Kaul A, Mandal S, Davidov O, Peddada SD. Analysis of microbiome data in the presence of excess zeros. Front Microbiol. 2017;8:2114. doi: 10.3389/fmicb.2017.02114 29163406 PMC5682008

[60] Jiang X, Dong S, Liu R, Huang M, Dong K, Ge J, et al. Effects of temperature, dissolved oxygen, and their interaction on the growth performance and condition of rainbow trout (Oncorhynchus mykiss). J Therm Biol. 2021;98:102928. doi: 10.1016/j.jtherbio.2021.102928 34016351

[61] Saoud I. Environmental impact of trout aquaculture on the Lebanon portion of the Assi River. World Aquacult Magazine. 2014.

[62] Lozupone C, Lladser ME, Knights D, Stombaugh J, Knight R. UniFrac: an effective distance metric for microbial community comparison. ISME J. 2011;5(2):169–72. doi: 10.1038/ismej.2010.133 20827291 PMC3105689

[63] Øverland M, Karlsson A, Mydland LT, Romarheim OH, Skrede A. Evaluation of Candida utilis, Kluyveromyces marxianus and Saccharomyces cerevisiae yeasts as protein sources in diets for Atlantic salmon (Salmo salar). Aquaculture. 2013;402–403:1–7. doi: 10.1016/j.aquaculture.2013.03.016

[64] Hauptman BS, Barrows FT, Block SS, Gibson Gaylord T, Paterson JA, Rawles SD, et al. Evaluation of grain distillers dried yeast as a fish meal substitute in practical-type diets of juvenile rainbow trout, Oncorhynchus mykiss. Aquaculture. 2014;432:7–14. doi: 10.1016/j.aquaculture.2014.03.026

[65] Hajimoradloo A. Effect of using yeast (Saccharomyces cerevisiae) as probiotic on growth parameters, survival and carcass quality in rainbow trout Oncorhynchus mykiss fry. Int Aquatic Res. 2009.

[66] Pough JD II, Hines IS, Layton AR, Smith SA, Huang H, O’Keefe SF, et al. Impact of Brewer’s Spent yeast supplemented diets on the growth rates and intestinal bacterial communities of farmed rainbow trout (Oncorhynchus mykiss). Aquacult Res. 2025;2025(1). doi: 10.1155/are/9305030

[67] Sealey WM, Gaylord TG, Barrows FT, Tomberlin JK, McGuire MA, Ross C, et al. Sensory analysis of rainbow trout, oncorhynchus mykiss, fed enriched Black soldier Fly Prepupae, Hermetia illucens. J World Aquaculture Soc. 2011;42(1):34–45. doi: 10.1111/j.1749-7345.2010.00441.x

[68] Zhou C, Yang S, Ka W, Gao P, Li Y, Long R, et al. Association of gut microbiota with metabolism in rainbow trout under acute heat stress. Front Microbiol. 2022;13:846336. doi: 10.3389/fmicb.2022.846336 35432278 PMC9007319

[69] Huyben D, Vidakovic A, Sundh H, Sundell K, Kiessling A, Lundh T. Haematological and intestinal health parameters of rainbow trout are influenced by dietary live yeast and increased water temperature. Fish Shellfish Immunol. 2019;89:525–36. doi: 10.1016/j.fsi.2019.04.047 30999040

[70] Zhou C-Q, Zhou P, Ren Y-L, Cao L-H, Wang J-L. Physiological response and miRNA-mRNA interaction analysis in the head kidney of rainbow trout exposed to acute heat stress. J Therm Biol. 2019;83:134–41. doi: 10.1016/j.jtherbio.2019.05.014 31331511

[71] Jones KA. Chemical requirements of feeding in rainbow trout, Oncorhynchus mykiss (Walbaum); Palatability studies on amino acids, amides, amines, alcohols, aldehydes, saccharides, and other compounds. J Fish Biol. 1990;37(3):413–23. doi: 10.1111/j.1095-8649.1990.tb05872.x

[72] Gülay A, Musovic S, Albrechtsen H-J, Al-Soud WA, Sørensen SJ, Smets BF. Ecological patterns, diversity and core taxa of microbial communities in groundwater-fed rapid gravity filters. ISME J. 2016;10(9):2209–22. doi: 10.1038/ismej.2016.16 26953601 PMC4989307

[73] Berg KA, Lyra C, Sivonen K, Paulin L, Suomalainen S, Tuomi P, et al. High diversity of cultivable heterotrophic bacteria in association with cyanobacterial water blooms. ISME J. 2009;3(3):314–25. doi: 10.1038/ismej.2008.110 19020559

[74] Huyben D, Sun L, Moccia R, Kiessling A, Dicksved J, Lundh T. Dietary live yeast and increased water temperature influence the gut microbiota of rainbow trout. J Appl Microbiol. 2018;124(6):1377–92. doi: 10.1111/jam.13738 29464844

[75] Suhr M, Fichtner-Grabowski F-T, Seibel H, Bang C, Franke A, Schulz C, et al. The microbiota knows: handling-stress and diet transform the microbial landscape in the gut content of rainbow trout in RAS. Anim Microbiome. 2023;5(1):33. doi: 10.1186/s42523-023-00253-9 37386608 PMC10311755

[76] Ringø E, Gatesoupe F-J. Lactic acid bacteria in fish: A review. Aquaculture. 1998;160(3–4):177–203. doi: 10.1016/s0044-8486(97)00299-8

[77] Hovda MB, Lunestad BT, Fontanillas R, Rosnes JT. Molecular characterisation of the intestinal microbiota of farmed Atlantic salmon (Salmo salar L.). Aquaculture. 2007;272(1–4):581–8. doi: 10.1016/j.aquaculture.2007.08.045

[78] Gatesoupe F-J. Updating the importance of lactic acid bacteria in fish farming: natural occurrence and probiotic treatments. J Mol Microbiol Biotechnol. 2008;14(1–3):107–14. doi: 10.1159/000106089 17957117

[79] Hines I, Marshall M, Smith S, Kuhn D, Stevens A. Systematic literature review identifying bacterial constituents in the core intestinal microbiome of rainbow trout (Oncorhynchus mykiss). Aquacult Fish Fisheries. 2023;3. doi: 10.1002/aff2.127

[80] Tsuchiya C, Sakata T, Sugita H. Novel ecological niche of Cetobacterium somerae, an anaerobic bacterium in the intestinal tracts of freshwater fish. Lett Appl Microbiol. 2008;46(1):43–8. doi: 10.1111/j.1472-765X.2007.02258.x 17944860

[81] Qi X, Zhang Y, Zhang Y, Luo F, Song K, Wang G, et al. Vitamin B12 produced by Cetobacterium somerae improves host resistance against pathogen infection through strengthening the interactions within gut microbiota. Microbiome. 2023;11(1):135. doi: 10.1186/s40168-023-01574-2 37322528 PMC10268390

[82] Janda JM, Abbott SL. The changing face of the family enterobacteriaceae (Order: “Enterobacterales”): New members, taxonomic issues, geographic expansion, and new diseases and disease syndromes. Clin Microbiol Rev. 2021;34(2):e00174–20. doi: 10.1128/CMR.00174-20 33627443 PMC8262773

[83] DeGruttola AK, Low D, Mizoguchi A, Mizoguchi E. Current understanding of dysbiosis in disease in human and animal models. Inflamm Bowel Dis. 2016;22(5):1137–50. doi: 10.1097/MIB.0000000000000750 27070911 PMC4838534

[84] Delghandi MR, El-Matbouli M, Menanteau-Ledouble S. Mycobacteriosis and infections with non-tuberculous mycobacteria in aquatic organisms: A review. Microorganisms. 2020;8(9):1368. doi: 10.3390/microorganisms8091368 32906655 PMC7564596

[85] Lescenko P, Matlova L, Dvorska L, Bartoš M, Vavra O, Navratil S. Mycobacterial infection in aquarium fish. Veterinarni Medicina. 2003;48:71–8. doi: 10.17221/5752-VETMED

[86] Prearo M, Zanoni RG, Campo Dall’Orto B, Pavoletti E, Florio D, Penati V, et al. Mycobacterioses: emerging pathologies in aquarium fish. Vet Res Commun. 2004;28 Suppl 1:315–7. doi: 10.1023/b:verc.0000045435.19522.af 15372986

[87] Yanong RPE, Pouder DB, Falkinham JO 3rd. Association of mycobacteria in recirculating aquaculture systems and mycobacterial disease in fish. J Aquat Anim Health. 2010;22(4):219–23. doi: 10.1577/H10-009.1 21413504

[88] Beran V, Matlova L, Dvorska L, Svastova P, Pavlik I. Distribution of mycobacteria in clinically healthy ornamental fish and their aquarium environment. J Fish Dis. 2006;29(7):383–93. doi: 10.1111/j.1365-2761.2006.00729.x 16866922

[89] Slany M, Makovcova J, Jezek P, Bodnarova M, Pavlik I. Relative prevalence of Mycobacterium marinum in fish collected from aquaria and natural freshwaters in central Europe. J Fish Dis. 2014;37(6):527–33. doi: 10.1111/jfd.12135 23952681

[90] Abdul Kari Z, Wee W, Mohamad Sukri SA, Che Harun H, Hanif Reduan MF, Irwan Khoo M, et al. Role of phytobiotics in relieving the impacts of Aeromonas hydrophila infection on aquatic animals: A mini-review. Front Vet Sci. 2022;9:1023784. doi: 10.3389/fvets.2022.1023784 36277060 PMC9582345

[91] Zepeda-Velázquez AP, Vega-Sánchez V, Salgado-Miranda C, Soriano-Vargas E. Histopathological findings in farmed rainbow trout (Oncorhynchus mykiss) naturally infected with 3 different Aeromonas species. Can J Vet Res. 2015;79(3):250–4. 26130859 PMC4445519

