# Supplementary material for: Using brewing industry byproducts to create feed additives for aquaculture-raised rainbow trout under thermal stress
Source: PLoS One. 2026 Nov 3;21(11):e0360302. doi: 10.1371/journal.pone.0360302 (PMC13730342; doi:10.1371/journal.pone.0360302)
Supplement: Supplementary file 1 [file pone.0360302.s005.docx]

**Supplementary Table 1. Summary of alpha diversity analyses of feed (mean ± standard error).**

|  | **Diet/Treatment** | **Shannon** | **Evenness** | **Observed ASVs** |
| --- | --- | --- | --- | --- |
| **Feed Samples** | **Control feed** | 6.83 ± 0.06^a^ | 0.86 ± 0.01^a^ | 238.50 ± 7.72^a^ |
|  | **Yeast** | 3.49 ± 0.13^b^ | 0.72 ± 0.03^a,b^ | 28.50 ± 0.71^b^ |
|  | **Old insect** | 3.62 ± 0.15^b^ | 0.58 ± 0.01^b^ | 75.00 ± 5.35^b^ |
|  | **New insect** | 4.69 ± 0.07^a,b^ | 0.69 ± 0.01^a,b^ | 111.00 ± 2.45^a,b^ |
|  | P-value^†^ | 0.008 | 0.007 | 0.007 |

^†^P-value of the one-way Kruskal-Wallis test.

^ab^Diversity metric within group significant at P < 0.05 following a one-way Kruskal-Wallis with Dunn’s post-hoc test.
