# Supplementary material for: Using brewing industry byproducts to create feed additives for aquaculture-raised rainbow trout under thermal stress
Source: PLoS One. 2026 Nov 3;21(11):e0360302. doi: 10.1371/journal.pone.0360302 (PMC13730342; doi:10.1371/journal.pone.0360302)
Supplement: Supplementary file 2 [file pone.0360302.s006.docx]

**Supplementary Table 2. Amplicon sequence variant relative abundance table (mean ± SEM) of trout intestinal samples ordered by the overall highest average read count of organisms based on Control(T2).**

| **Phylogeny** | | | **Relative Abundance (%)** | | | | | |
| --- | --- | --- | --- | --- | --- | --- | --- | --- |
| **Phylum** | **Family** | **Organism** | **Control(T1)** | **Control(T2)** | **Control(ND)** | **BSY2** | **BSF3** | **Mix** |
| Actinobacteriota | *Mycobacteriaceae* | ***Mycobacterium* sp.** | 0.00 ± 0.00 | 44.21 ± 1.34 | 2.22 ± 0.14 | 28.96 ± 1.02 | 26.98 ± 1.52 | 40.99 ± 1.25 |
| Unassigned Phyla | *Unassigned* | **Unassigned** | 23.19 ± 2.72 | 12.11 ± 1.06 | 14.14 ± 1.19 | 17.3 ± 1.32 | 4.47 ± 0.48 | 16.41 ± 1.42 |
| Firmicutes | *Mycoplasmataceae* | ***Mycoplasma* sp.** | 0.00 ± 0.00 | 10.32 ± 1.27 | 3.51 ± 0.34 | 20.15 ± 1.3 | 13.12 ± 1.49 | 8.06 ± 0.53 |
| Proteobacteria | *Rhizobiales Incertae Sedis* | ***Rhizobiales Incertae Sedis*** | 0.00 ± 0.00 | 7.86 ± 0.35 | 0.24 ± 0.01 | 4.22 ± 0.16 | 4.43 ± 0.27 | 8.88 ± 0.31 |
| Proteobacteria | *Xanthobacteraceae* | ***Ancylobacter* sp.** | 0.00 ± 0.00 | 7.14 ± 0.27 | 0.08 ± 0.00 | 5.31 ± 0.16 | 5.45 ± 0.32 | 8.07 ± 0.28 |
| Fusobacteriota | *Fusobacteriaceae* | ***Cetobacterium* sp.** | 2.42 ± 0.27 | 4.75 ± 0.29 | 54.72 ± 1.74 | 6.72 ± 0.41 | 32.73 ± 1.78 | 4.49 ± 0.35 |
| Proteobacteria | *Oxalobacteraceae* | ***Undibacterium* sp.** | 0.00 ± 0.00 | 2.38 ± 0.30 | 0.00 ± 0.00 | 0.15 ± 0.01 | 0.00 ± 0.00 | 0.00 ± 0.00 |
| Proteobacteria | *Reyranellaceae* | ***Reyranella* sp.** | 0.00 ± 0.00 | 2.06 ± 0.22 | 0.02 ± 0.00 | 0.69 ± 0.06 | 0.41 ± 0.03 | 0.13 ± 0.01 |
| Proteobacteria | *Sphingomonadaceae* | ***Novosphingobium* sp.** | 0.00 ± 0.00 | 0.83 ± 0.10 | 0.02 ± 0.00 | 2.91 ± 0.36 | 0.02 ± 0.00 | 0.00 ± 0.00 |
| Proteobacteria | *Comamonadaceae* | ***Comamonadaceae*** | 0.08 ± 0.01 | 0.72 ± 0.06 | 0.04 ± 0.00 | 0.46 ± 0.03 | 0.65 ± 0.08 | 0.19 ± 0.02 |
| Proteobacteria | *Aeromonadaceae* | ***Aeromonas* sp.** | 0.00 ± 0.00 | 0.57 ± 0.03 | 12.35 ± 0.96 | 8.09 ± 0.98 | 4.71 ± 0.35 | 7.94 ± 0.94 |
| Firmicutes | *Bacillaceae* | ***Bacillus* sp.** | 0.00 ± 0.00 | 0.38 ± 0.02 | 0.27 ± 0.02 | 0.12 ± 0.01 | 0.14 ± 0.01 | 0.35 ± 0.02 |
| Firmicutes | *Clostridiaceae* | ***Clostridium sensu stricto 1* sp.** | 13.81 ± 1.39 | 0.30 ± 0.03 | 0.62 ± 0.07 | 0.13 ± 0.01 | 1.10 ± 0.14 | 0.11 ± 0.01 |
| Proteobacteria | *Oxalobacteraceae* | ***Janthinobacterium* sp.** | 0.00 ± 0.00 | 0.30 ± 0.04 | 0.00 ± 0.00 | 0.19 ± 0.02 | 0.00 ± 0.00 | 0.00 ± 0.00 |
| Planctomycetota | *Isosphaeraceae* | ***Tundrisphaera* sp.** | 0.00 ± 0.00 | 0.29 ± 0.01 | 0.00 ± 0.00 | 0.05 ± 0.00 | 0.08 ± 0.01 | 0.24 ± 0.01 |
| Proteobacteria | *Sphingomonadaceae* | ***Sphingopyxis* sp.** | 0.00 ± 0.00 | 0.29 ± 0.02 | 0.00 ± 0.00 | 0.26 ± 0.03 | 1.64 ± 0.20 | 0.00 ± 0.00 |
| Proteobacteria | *Legionellaceae* | ***Legionella* sp.** | 0.00 ± 0.00 | 0.28 ± 0.01 | 0.00 ± 0.00 | 0.13 ± 0.01 | 0.16 ± 0.01 | 0.29 ± 0.01 |
| Proteobacteria | *Rhizobiaceae* | ***Shinella* sp.** | 0.00 ± 0.00 | 0.28 ± 0.02 | 0.01 ± 0.00 | 0.28 ± 0.03 | 0.00 ± 0.00 | 0.00 ± 0.00 |
| Actinobacteriota | PeM15 | **PeM15 sp.** | 0.00 ± 0.00 | 0.25 ± 0.01 | 0.00 ± 0.00 | 0.08 ± 0.00 | 0.08 ± 0.01 | 0.18 ± 0.01 |
| Proteobacteria | *Enterobacteriaceae* | ***Escherichia-Shigella* sp.** | 0.00 ± 0.00 | 0.24 ± 0.03 | 0.00 ± 0.00 | 0.00 ± 0.00 | 0.00 ± 0.00 | 0.00 ± 0.00 |
| Bacteroidota | *Flavobacteriaceae* | ***Flavobacterium* sp.** | 0.00 ± 0.00 | 0.21 ± 0.02 | 0.00 ± 0.00 | 0.15 ± 0.02 | 0.00 ± 0.00 | 0.00 ± 0.00 |
| Verrucomicrobiota | *Simkaniaceae* | ***Simkaniaceae*** | 0.00 ± 0.00 | 0.20 ± 0.01 | 0.00 ± 0.00 | 0.16 ± 0.01 | 0.08 ± 0.00 | 0.21 ± 0.01 |
| Proteobacteria | *Diplorickettsiaceae* | ***Diplorickettsiaceae*** | 0.00 ± 0.00 | 0.15 ± 0.00 | 0.00 ± 0.00 | 0.05 ± 0.00 | 0.12 ± 0.01 | 0.22 ± 0.01 |
| Proteobacteria | *Pseudomonadaceae* | ***Pseudomonas* sp.** | 0.00 ± 0.00 | 0.14 ± 0.02 | 0.00 ± 0.00 | 0.00 ± 0.00 | 0.00 ± 0.00 | 0.00 ± 0.00 |
| Proteobacteria | *Enterobacteriaceae* | ***Enterobacteriaceae*** | 0.00 ± 0.00 | 0.13 ± 0.01 | 10.62 ± 0.94 | 0.28 ± 0.02 | 0.35 ± 0.03 | 0.11 ± 0.01 |
| Proteobacteria | *Acetobacteraceae* | ***Acidisphaera* sp.** | 0.00 ± 0.00 | 0.11 ± 0.01 | 0.00 ± 0.00 | 0.00 ± 0.00 | 0.04 ± 0.00 | 0.00 ± 0.00 |
| Firmicutes | *Peptostreptococcaceae* | ***Peptostreptococcus* sp.** | 4.46 ± 0.26 | 0.11 ± 0.01 | 0.00 ± 0.00 | 0.00 ± 0.00 | 0.00 ± 0.00 | 0.00 ± 0.00 |
| Proteobacteria | *Caedibacteraceae* | ***Caedimonas varicaedens*** | 0.00 ± 0.00 | 0.11 ± 0.01 | 0.00 ± 0.00 | 0.07 ± 0.01 | 0.00 ± 0.00 | 0.00 ± 0.00 |
| Proteobacteria | *Rhodobacteraceae* | ***Rhodobacter* sp.** | 0.00 ± 0.00 | 0.10 ± 0.01 | 0.00 ± 0.00 | 0.07 ± 0.01 | 0.00 ± 0.00 | 0.00 ± 0.00 |
| Proteobacteria | *Beijerinckiaceae* | ***Methylobacterium-Methylorubrum* sp.** | 0.00 ± 0.00 | 0.10 ± 0.01 | 0.01 ± 0.00 | 0.13 ± 0.01 | 0.00 ± 0.00 | 0.00 ± 0.00 |
| Proteobacteria | *Chromobacteriaceae* | ***Vogesella fluminis*** | 0.00 ± 0.00 | 0.10 ± 0.01 | 0.00 ± 0.00 | 0.00 ± 0.00 | 0.00 ± 0.00 | 0.00 ± 0.00 |
| Proteobacteria | *Xanthomonadaceae* | ***Stenotrophomonas* sp.** | 0.00 ± 0.00 | 0.10 ± 0.01 | 0.00 ± 0.00 | 0.00 ± 0.00 | 0.28 ± 0.03 | 0.00 ± 0.00 |
| Proteobacteria | *Xanthobacteraceae* | ***Xanthobacteraceae*** | 0.00 ± 0.00 | 0.10 ± 0.01 | 0.00 ± 0.00 | 0.16 ± 0.02 | 0.10 ± 0.01 | 0.00 ± 0.00 |
| Proteobacteria | *Solimonadaceae* | ***Nevskia* sp.** | 0.00 ± 0.00 | 0.09 ± 0.01 | 0.00 ± 0.00 | 0.00 ± 0.00 | 0.00 ± 0.00 | 0.00 ± 0.00 |
| Planctomycetota | *Pirellulaceae* | **Pir4 lineage sp.** | 0.00 ± 0.00 | 0.09 ± 0.01 | 0.00 ± 0.00 | 0.07 ± 0.01 | 0.03 ± 0.00 | 0.05 ± 0.00 |
| Proteobacteria | *Comamonadaceae* | ***Rubrivivax* sp.** | 0.00 ± 0.00 | 0.08 ± 0.01 | 0.00 ± 0.00 | 0.00 ± 0.00 | 0.00 ± 0.00 | 0.00 ± 0.00 |
| Proteobacteria | SC-I-84 | **SC-I-84 sp.** | 0.00 ± 0.00 | 0.08 ± 0.01 | 0.00 ± 0.00 | 0.00 ± 0.00 | 0.00 ± 0.00 | 0.00 ± 0.00 |
| Bdellovibrionota | 0319-6G20 | **0319-6G20 sp.** | 0.00 ± 0.00 | 0.07 ± 0.01 | 0.00 ± 0.00 | 0.06 ± 0.01 | 0.00 ± 0.00 | 0.01 ± 0.00 |
| Proteobacteria | *Chromobacteriaceae* | ***Vogesella* sp.** | 0.00 ± 0.00 | 0.07 ± 0.01 | 0.00 ± 0.00 | 0.00 ± 0.00 | 0.00 ± 0.00 | 0.00 ± 0.00 |
| Planctomycetota | *Pirellulaceae* | ***Pirellulaceae*** | 0.00 ± 0.00 | 0.07 ± 0.00 | 0.00 ± 0.00 | 0.00 ± 0.00 | 0.02 ± 0.00 | 0.04 ± 0.00 |
| Proteobacteria | *Moraxellaceae* | ***Acinetobacter* sp.** | 0.94 ± 0.08 | 0.07 ± 0.01 | 0.00 ± 0.00 | 0.07 ± 0.01 | 0.03 ± 0.00 | 0.00 ± 0.00 |
| Proteobacteria | *Alcaligenaceae* | ***Alcaligenaceae*** | 0.00 ± 0.00 | 0.06 ± 0.01 | 0.00 ± 0.00 | 0.00 ± 0.00 | 0.00 ± 0.00 | 0.00 ± 0.00 |
| Proteobacteria | *Rhodocyclaceae* | ***Methyloversatilis* sp.** | 0.00 ± 0.00 | 0.06 ± 0.01 | 0.00 ± 0.00 | 0.00 ± 0.00 | 0.00 ± 0.00 | 0.00 ± 0.00 |
| Proteobacteria | *Xanthobacteraceae* | ***Bradyrhizobium* sp.** | 0.00 ± 0.00 | 0.06 ± 0.00 | 0.00 ± 0.00 | 0.05 ± 0.00 | 0.16 ± 0.02 | 0.02 ± 0.00 |
| Firmicutes | *Lactobacillaceae* | ***Lactobacillus* sp.** | 0.26 ± 0.02 | 0.06 ± 0.01 | 0.00 ± 0.00 | 0.00 ± 0.00 | 0.00 ± 0.00 | 0.00 ± 0.00 |
| Firmicutes | *Bacillaceae* | ***Cerasibacillus* sp.** | 0.00 ± 0.00 | 0.05 ± 0.00 | 0.03 ± 0.00 | 0.27 ± 0.03 | 0.00 ± 0.00 | 0.00 ± 0.00 |
| Bacteroidota | *Flavobacteriaceae* | **Flavobacteriia bacterium** | 0.00 ± 0.00 | 0.05 ± 0.01 | 0.00 ± 0.00 | 0.00 ± 0.00 | 0.00 ± 0.00 | 0.00 ± 0.00 |
| Proteobacteria | *Comamonadaceae* | ***Variovorax* sp.** | 0.00 ± 0.00 | 0.05 ± 0.01 | 0.00 ± 0.00 | 0.00 ± 0.00 | 0.00 ± 0.00 | 0.00 ± 0.00 |
| Proteobacteria | *Beijerinckiaceae* | ***Bosea* sp.** | 0.00 ± 0.00 | 0.05 ± 0.00 | 0.00 ± 0.00 | 0.06 ± 0.01 | 0.09 ± 0.01 | 0.03 ± 0.00 |
| Actinobacteriota | *Microbacteriaceae* | ***Galbitalea* sp.** | 0.00 ± 0.00 | 0.05 ± 0.00 | 0.00 ± 0.00 | 0.00 ± 0.00 | 0.02 ± 0.00 | 0.05 ± 0.00 |
| Spirochaetota | *Brevinemataceae* | **uncultured spirochete** | 15.72 ± 1.69 | 0.00 ± 0.00 | 0.00 ± 0.00 | 0.10 ± 0.01 | 0.00 ± 0.00 | 0.00 ± 0.00 |
| Firmicutes | *Streptococcaceae* | ***Streptococcus* sp.** | 13.48 ± 0.84 | 0.00 ± 0.00 | 0.00 ± 0.00 | 0.00 ± 0.00 | 0.00 ± 0.00 | 0.00 ± 0.00 |
| Firmicutes | *Streptococcaceae* | ***Streptococcus dysgalactiae*** | 8.21 ± 0.54 | 0.00 ± 0.00 | 0.00 ± 0.00 | 0.00 ± 0.00 | 0.00 ± 0.00 | 0.00 ± 0.00 |
| Firmicutes | *Streptococcaceae* | ***Streptococcus agalactiae*** | 2.56 ± 0.15 | 0.00 ± 0.00 | 0.00 ± 0.00 | 0.00 ± 0.00 | 0.00 ± 0.00 | 0.00 ± 0.00 |
| Firmicutes | *Streptococcaceae* | ***Lactococcus lactis*** | 1.88 ± 0.10 | 0.00 ± 0.00 | 0.00 ± 0.00 | 0.00 ± 0.00 | 0.00 ± 0.00 | 0.00 ± 0.00 |
| Firmicutes | *Streptococcaceae* | ***Streptococcus henryi*** | 1.23 ± 0.08 | 0.00 ± 0.00 | 0.00 ± 0.00 | 0.00 ± 0.00 | 0.00 ± 0.00 | 0.00 ± 0.00 |
| Proteobacteria | *Yersiniaceae* | ***Yersiniaceae*** | 0.78 ± 0.13 | 0.00 ± 0.00 | 0.13 ± 0.01 | 0.00 ± 0.00 | 0.00 ± 0.00 | 0.04 ± 0.01 |
| Firmicutes | *Peptostreptococcales-Tissierellales* | ***Peptoniphilus indolicus*** | 0.69 ± 0.05 | 0.00 ± 0.00 | 0.00 ± 0.00 | 0.00 ± 0.00 | 0.00 ± 0.00 | 0.00 ± 0.00 |
| Firmicutes | *Carnobacteriaceae* | ***Granulicatella* sp.** | 0.64 ± 0.04 | 0.00 ± 0.00 | 0.00 ± 0.00 | 0.00 ± 0.00 | 0.00 ± 0.00 | 0.00 ± 0.00 |
| Firmicutes | *Enterococcaceae* | ***Enterococcus* sp.** | 0.55 ± 0.03 | 0.00 ± 0.00 | 0.00 ± 0.00 | 0.00 ± 0.00 | 0.00 ± 0.00 | 0.00 ± 0.00 |
| Firmicutes | *Peptococcaceae* | ***Peptococcus* sp.** | 0.55 ± 0.03 | 0.00 ± 0.00 | 0.00 ± 0.00 | 0.00 ± 0.00 | 0.00 ± 0.00 | 0.00 ± 0.00 |
| Firmicutes | *Peptostreptococcales-Tissierellales* | ***Gallicola* sp.** | 0.54 ± 0.03 | 0.00 ± 0.00 | 0.00 ± 0.00 | 0.00 ± 0.00 | 0.00 ± 0.00 | 0.00 ± 0.00 |
| Firmicutes | *Streptococcaceae* | ***Lactococcus garvieae*** | 0.53 ± 0.03 | 0.00 ± 0.00 | 0.00 ± 0.00 | 0.00 ± 0.00 | 0.00 ± 0.00 | 0.00 ± 0.00 |
| Actinobacteriota | *Coriobacteriales Incertae Sedis* | ***Raoultibacter* sp.** | 0.44 ± 0.03 | 0.00 ± 0.00 | 0.00 ± 0.00 | 0.00 ± 0.00 | 0.00 ± 0.00 | 0.00 ± 0.00 |
| Firmicutes | *Streptococcaceae* | ***Streptococcus iniae*** | 0.40 ± 0.02 | 0.00 ± 0.00 | 0.00 ± 0.00 | 0.00 ± 0.00 | 0.00 ± 0.00 | 0.00 ± 0.00 |
| Firmicutes | *Streptococcaceae* | ***Streptococcus minor*** | 0.31 ± 0.03 | 0.00 ± 0.00 | 0.00 ± 0.00 | 0.00 ± 0.00 | 0.00 ± 0.00 | 0.00 ± 0.00 |
| Firmicutes | *Streptococcaceae* | ***Streptococcus suis*** | 0.31 ± 0.02 | 0.00 ± 0.00 | 0.00 ± 0.00 | 0.00 ± 0.00 | 0.00 ± 0.00 | 0.00 ± 0.00 |
| Firmicutes | *Lachnospiraceae* | ***Lachnospiraceae*** | 0.29 ± 0.02 | 0.00 ± 0.00 | 0.00 ± 0.00 | 0.00 ± 0.00 | 0.00 ± 0.00 | 0.00 ± 0.00 |
| Firmicutes | *Peptostreptococcales-Tissierellales* | **swine effluent** | 0.26 ± 0.02 | 0.00 ± 0.00 | 0.00 ± 0.00 | 0.00 ± 0.00 | 0.00 ± 0.00 | 0.00 ± 0.00 |
| Firmicutes | *Streptococcaceae* | ***Lactococcus raffinolactis*** | 0.25 ± 0.01 | 0.00 ± 0.00 | 0.00 ± 0.00 | 0.00 ± 0.00 | 0.02 ± 0.00 | 0.02 ± 0.00 |
| Actinobacteriota | *Atopobiaceae* | ***Atopobiaceae*** | 0.22 ± 0.01 | 0.00 ± 0.00 | 0.00 ± 0.00 | 0.00 ± 0.00 | 0.00 ± 0.00 | 0.00 ± 0.00 |
| Firmicutes | *Peptostreptococcales-Tissierellales* | ***Peptoniphilus methioninivorax*** | 0.21 ± 0.01 | 0.00 ± 0.00 | 0.00 ± 0.00 | 0.00 ± 0.00 | 0.00 ± 0.00 | 0.00 ± 0.00 |
| Firmicutes | *Streptococcaceae* | ***Streptococcus parauberis*** | 0.20 ± 0.01 | 0.00 ± 0.00 | 0.00 ± 0.00 | 0.00 ± 0.00 | 0.00 ± 0.00 | 0.00 ± 0.00 |
| Firmicutes | *Streptococcaceae* | ***Streptococcus gallinaceus*** | 0.18 ± 0.01 | 0.00 ± 0.00 | 0.00 ± 0.00 | 0.00 ± 0.00 | 0.00 ± 0.00 | 0.00 ± 0.00 |
| Firmicutes | *Peptostreptococcales-Tissierellales* | **swine manure** | 0.18 ± 0.01 | 0.00 ± 0.00 | 0.00 ± 0.00 | 0.00 ± 0.00 | 0.00 ± 0.00 | 0.00 ± 0.00 |
| Firmicutes | *Streptococcaceae* | ***Lactococcus* sp.** | 0.16 ± 0.01 | 0.00 ± 0.00 | 0.00 ± 0.00 | 0.00 ± 0.00 | 0.00 ± 0.00 | 0.00 ± 0.00 |
| Firmicutes | *Peptostreptococcales-Tissierellales* | ***Peptostreptococcales-Tissierellales*** | 0.14 ± 0.02 | 0.00 ± 0.00 | 0.00 ± 0.00 | 0.00 ± 0.00 | 0.00 ± 0.00 | 0.00 ± 0.00 |
| Firmicutes | *Streptococcaceae* | ***Streptococcus pluranimalium*** | 0.12 ± 0.01 | 0.00 ± 0.00 | 0.00 ± 0.00 | 0.00 ± 0.00 | 0.00 ± 0.00 | 0.00 ± 0.00 |
| Firmicutes | *Lachnospiraceae* | ***Frisingicoccus* sp.** | 0.11 ± 0.01 | 0.00 ± 0.00 | 0.00 ± 0.00 | 0.00 ± 0.00 | 0.00 ± 0.00 | 0.00 ± 0.00 |
| Firmicutes | *Lachnospiraceae* | ***Catenibacillus* sp.** | 0.11 ± 0.01 | 0.00 ± 0.00 | 0.00 ± 0.00 | 0.00 ± 0.00 | 0.00 ± 0.00 | 0.00 ± 0.00 |
| Firmicutes | *Streptococcaceae* | ***Streptococcus* sp.** | 0.00 ± 0.00 | 0.10 ± 0.01 | 0.00 ± 0.00 | 0.00 ± 0.00 | 0.00 ± 0.00 | 0.00 ± 0.00 |
| Actinobacteriota | *Corynebacteriaceae* | ***Corynebacterium* sp.** | 0.09 ± 0.01 | 0.00 ± 0.00 | 0.01 ± 0.00 | 0.00 ± 0.00 | 0.03 ± 0.00 | 0.04 ± 0.00 |
| Firmicutes | *Peptostreptococcaceae* | ***Romboutsia* sp.** | 0.09 ± 0.01 | 0.00 ± 0.00 | 0.00 ± 0.00 | 0.00 ± 0.00 | 0.00 ± 0.00 | 0.00 ± 0.00 |
| Firmicutes | *Order  Lactobacillales* | **Order  Lactobacillales** | 0.08 ± 0.01 | 0.00 ± 0.00 | 0.00 ± 0.00 | 0.00 ± 0.00 | 0.00 ± 0.00 | 0.00 ± 0.00 |
| Fusobacteriota | *Fusobacteriaceae* | **Fusobacteriaceae bacterium** | 0.08 ± 0.00 | 0.00 ± 0.00 | 0.00 ± 0.00 | 0.00 ± 0.00 | 0.00 ± 0.00 | 0.00 ± 0.00 |
| Firmicutes | *Vagococcaceae* | ***Vagococcus teuberi*** | 0.08 ± 0.00 | 0.00 ± 0.00 | 0.00 ± 0.00 | 0.00 ± 0.00 | 0.00 ± 0.00 | 0.00 ± 0.00 |
| Proteobacteria | *Vibrionaceae* | ***Photobacterium* sp.** | 0.07 ± 0.01 | 0.00 ± 0.00 | 0.00 ± 0.00 | 0.00 ± 0.00 | 0.00 ± 0.00 | 0.00 ± 0.00 |
| Firmicutes | *Peptostreptococcales-Tissierellales* | ***Helcococcus* sp.** | 0.07 ± 0.00 | 0.00 ± 0.00 | 0.00 ± 0.00 | 0.00 ± 0.00 | 0.00 ± 0.00 | 0.00 ± 0.00 |
| Firmicutes | *Lachnospiraceae* | ***Blautia glucerasea*** | 0.07 ± 0.01 | 0.00 ± 0.00 | 0.00 ± 0.00 | 0.00 ± 0.00 | 0.00 ± 0.00 | 0.00 ± 0.00 |
| Firmicutes | *Staphylococcaceae* | ***Staphylococcus* sp.** | 0.07 ± 0.00 | 0.00 ± 0.00 | 0.01 ± 0.00 | 0.13 ± 0.01 | 0.00 ± 0.00 | 0.00 ± 0.00 |
| Firmicutes | *Veillonellaceae* | ***Veillonella* sp.** | 0.07 ± 0.00 | 0.00 ± 0.00 | 0.00 ± 0.00 | 0.00 ± 0.00 | 0.00 ± 0.00 | 0.00 ± 0.00 |
| Firmicutes | *Peptostreptococcales-Tissierellales* | ***Peptoniphilus stercorisuis*** | 0.07 ± 0.00 | 0.00 ± 0.00 | 0.00 ± 0.00 | 0.00 ± 0.00 | 0.00 ± 0.00 | 0.00 ± 0.00 |
| Firmicutes | *Vagococcaceae* | ***Vagococcus fluvialis*** | 0.07 ± 0.00 | 0.00 ± 0.00 | 0.00 ± 0.00 | 0.00 ± 0.00 | 0.00 ± 0.00 | 0.00 ± 0.00 |
| Proteobacteria | *Hafniaceae* | ***Hafnia-Obesumbacterium* sp.** | 0.00 ± 0.00 | 0.00 ± 0.00 | 0.35 ± 0.03 | 0.00 ± 0.00 | 0.00 ± 0.00 | 0.21 ± 0.03 |
| Firmicutes | *Clostridiaceae* | ***Clostridium gasigenes*** | 0.00 ± 0.00 | 0.00 ± 0.00 | 0.26 ± 0.03 | 0.08 ± 0.01 | 1.08 ± 0.13 | 0.79 ± 0.10 |
| Firmicutes | *Clostridiaceae* | ***Clostridium amazonense*** | 0.00 ± 0.00 | 0.00 ± 0.00 | 0.07 ± 0.01 | 0.00 ± 0.00 | 0.00 ± 0.00 | 0.84 ± 0.10 |
| Firmicutes | *Clostridiaceae* | ***Clostridium botulinum*** | 0.00 ± 0.00 | 0.00 ± 0.00 | 0.04 ± 0.01 | 0.00 ± 0.00 | 0.00 ± 0.00 | 0.00 ± 0.00 |
| Cyanobacteria | *Sericytochromatia* | ***Sericytochromatia* sp.** | 0.00 ± 0.00 | 0.00 ± 0.00 | 0.03 ± 0.00 | 0.00 ± 0.00 | 0.00 ± 0.00 | 0.00 ± 0.00 |
| Proteobacteria | *Sphingomonadaceae* | ***Sphingobium* sp.** | 0.00 ± 0.00 | 0.00 ± 0.00 | 0.02 ± 0.00 | 0.06 ± 0.01 | 0.00 ± 0.00 | 0.00 ± 0.00 |
| Chloroflexi | RBG-13-54-9 | **RBG-13-54-9 sp.** | 0.00 ± 0.00 | 0.00 ± 0.00 | 0.01 ± 0.00 | 0.00 ± 0.00 | 0.00 ± 0.00 | 0.00 ± 0.00 |
| Firmicutes | *Paenibacillaceae* | ***Paenibacillus odorifer*** | 0.00 ± 0.00 | 0.00 ± 0.00 | 0.01 ± 0.00 | 0.09 ± 0.01 | 0.00 ± 0.00 | 0.19 ± 0.02 |
| Firmicutes | *Bacillaceae* | ***Oceanobacillus caeni*** | 0.00 ± 0.00 | 0.00 ± 0.00 | 0.01 ± 0.00 | 0.16 ± 0.02 | 0.00 ± 0.00 | 0.00 ± 0.00 |
| Proteobacteria | *Oxalobacteraceae* | ***Massilia* sp.** | 0.00 ± 0.00 | 0.00 ± 0.00 | 0.01 ± 0.00 | 0.00 ± 0.00 | 0.00 ± 0.00 | 0.00 ± 0.00 |
| Desulfobacterota | *Desulfovibrionaceae* | ***Desulfovibrionaceae*** | 0.00 ± 0.00 | 0.00 ± 0.00 | 0.01 ± 0.00 | 0.00 ± 0.00 | 0.00 ± 0.00 | 0.00 ± 0.00 |
| Bacteroidota | *Weeksellaceae* | ***Cloacibacterium* sp.** | 0.00 ± 0.00 | 0.00 ± 0.00 | 0.01 ± 0.00 | 0.00 ± 0.00 | 0.03 ± 0.00 | 0.13 ± 0.02 |
| Firmicutes | *Bacillaceae* | ***Bacillus oleronius*** | 0.00 ± 0.00 | 0.00 ± 0.00 | 0.01 ± 0.00 | 0.00 ± 0.00 | 0.00 ± 0.00 | 0.00 ± 0.00 |
| Verrucomicrobiota | *Parachlamydiaceae* | ***Parachlamydiaceae*** | 0.00 ± 0.00 | 0.00 ± 0.00 | 0.01 ± 0.00 | 0.00 ± 0.00 | 0.00 ± 0.00 | 0.02 ± 0.00 |
| Proteobacteria | *Sutterellaceae* | ***Sutterellaceae*** | 0.00 ± 0.00 | 0.00 ± 0.00 | 0.01 ± 0.00 | 0.05 ± 0.00 | 0.00 ± 0.00 | 0.00 ± 0.00 |
| Firmicutes | *Clostridiaceae* | ***Clostridium argentinense*** | 0.00 ± 0.00 | 0.00 ± 0.00 | 0.01 ± 0.00 | 0.00 ± 0.00 | 0.00 ± 0.00 | 0.00 ± 0.00 |
| Proteobacteria | *Chromobacteriaceae* | ***Aquitalea* sp.** | 0.00 ± 0.00 | 0.00 ± 0.00 | 0.00 ± 0.00 | 0.00 ± 0.00 | 0.00 ± 0.00 | 0.00 ± 0.00 |
| Proteobacteria | *Comamonadaceae* | ***Hydrogenophaga* sp.** | 0.00 ± 0.00 | 0.00 ± 0.00 | 0.00 ± 0.00 | 0.00 ± 0.00 | 0.07 ± 0.01 | 0.00 ± 0.00 |
| Actinobacteriota | *Actinomycetaceae* | **bacterium NLAE-zl-C91** | 0.00 ± 0.00 | 0.00 ± 0.00 | 0.00 ± 0.00 | 0.00 ± 0.00 | 0.00 ± 0.00 | 0.00 ± 0.00 |
| Firmicutes | *Bacillaceae* | ***Pseudogracilibacillus* sp.** | 0.00 ± 0.00 | 0.00 ± 0.00 | 0.00 ± 0.00 | 0.00 ± 0.00 | 0.00 ± 0.00 | 0.00 ± 0.00 |
| Deinococcota | *Thermaceae* | ***Meiothermus* sp.** | 0.00 ± 0.00 | 0.00 ± 0.00 | 0.00 ± 0.00 | 0.00 ± 0.00 | 0.00 ± 0.00 | 0.00 ± 0.00 |
| Firmicutes | *Paenibacillaceae* | ***Paenibacillus cookii*** | 0.00 ± 0.00 | 0.00 ± 0.00 | 0.00 ± 0.00 | 0.00 ± 0.00 | 0.00 ± 0.00 | 0.00 ± 0.00 |
| Planctomycetota | *Rubinisphaeraceae* | **uncultured planctomycete** | 0.00 ± 0.00 | 0.00 ± 0.00 | 0.00 ± 0.00 | 0.00 ± 0.00 | 0.01 ± 0.00 | 0.04 ± 0.00 |
| Bacteroidota | *Chitinophagaceae* | ***Sediminibacterium* sp.** | 0.00 ± 0.00 | 0.00 ± 0.00 | 0.00 ± 0.00 | 0.00 ± 0.00 | 0.00 ± 0.00 | 0.00 ± 0.00 |
| Actinobacteriota | *Actinomycetaceae* | **uncultured Actinomycetales** | 0.00 ± 0.00 | 0.00 ± 0.00 | 0.00 ± 0.00 | 0.00 ± 0.00 | 0.00 ± 0.00 | 0.00 ± 0.00 |
| Verrucomicrobiota | *Rubritaleaceae* | ***Luteolibacter* sp.** | 0.00 ± 0.00 | 0.00 ± 0.00 | 0.00 ± 0.00 | 0.13 ± 0.01 | 0.01 ± 0.00 | 0.01 ± 0.00 |
| Proteobacteria | *Pseudomonadaceae* | ***Azotobacter* sp.** | 0.00 ± 0.00 | 0.00 ± 0.00 | 0.00 ± 0.00 | 0.11 ± 0.01 | 0.15 ± 0.02 | 0.00 ± 0.00 |
| Verrucomicrobiota | *Rubritaleaceae* | ***Verrucomicrobium* sp.** | 0.00 ± 0.00 | 0.00 ± 0.00 | 0.00 ± 0.00 | 0.08 ± 0.01 | 0.01 ± 0.00 | 0.00 ± 0.00 |
| Proteobacteria | *Rhizobiaceae* | ***Rhizobiaceae*** | 0.00 ± 0.00 | 0.00 ± 0.00 | 0.00 ± 0.00 | 0.07 ± 0.01 | 0.00 ± 0.00 | 0.01 ± 0.00 |
| Proteobacteria | *Rhodocyclaceae* | ***Azospira* sp.** | 0.00 ± 0.00 | 0.00 ± 0.00 | 0.00 ± 0.00 | 0.07 ± 0.01 | 0.01 ± 0.00 | 0.00 ± 0.00 |
| Firmicutes | *Peptostreptococcaceae* | ***Paraclostridium* sp.** | 0.00 ± 0.00 | 0.00 ± 0.00 | 0.00 ± 0.00 | 0.05 ± 0.01 | 0.00 ± 0.00 | 0.00 ± 0.00 |
| Chloroflexi | JG30-KF-CM45 | **JG30-KF-CM45 sp.** | 0.00 ± 0.00 | 0.00 ± 0.00 | 0.00 ± 0.00 | 0.05 ± 0.01 | 0.00 ± 0.00 | 0.02 ± 0.00 |
| Dependentiae | *Vermiphilaceae* | ***Vermiphilaceae* sp.** | 0.00 ± 0.00 | 0.00 ± 0.00 | 0.00 ± 0.00 | 0.05 ± 0.00 | 0.05 ± 0.00 | 0.04 ± 0.00 |
| Bacteroidota | *Sphingobacteriaceae* | ***Pedobacter* sp.** | 0.00 ± 0.00 | 0.00 ± 0.00 | 0.00 ± 0.00 | 0.05 ± 0.01 | 0.00 ± 0.00 | 0.00 ± 0.00 |
| Firmicutes | *Carnobacteriaceae* | ***Alloiococcus* sp.** | 0.00 ± 0.00 | 0.00 ± 0.00 | 0.00 ± 0.00 | 0.04 ± 0.00 | 0.00 ± 0.00 | 0.00 ± 0.00 |
| Proteobacteria | *Hyphomicrobiaceae* | ***Hyphomicrobium* sp.** | 0.00 ± 0.00 | 0.00 ± 0.00 | 0.00 ± 0.00 | 0.00 ± 0.00 | 0.23 ± 0.03 | 0.00 ± 0.00 |
| Proteobacteria | *Xanthomonadaceae* | ***Pseudoxanthomonas* sp.** | 0.00 ± 0.00 | 0.00 ± 0.00 | 0.00 ± 0.00 | 0.00 ± 0.00 | 0.20 ± 0.02 | 0.00 ± 0.00 |
| Proteobacteria | *Caulobacteraceae* | ***Phenylobacterium* sp.** | 0.00 ± 0.00 | 0.00 ± 0.00 | 0.00 ± 0.00 | 0.00 ± 0.00 | 0.08 ± 0.01 | 0.00 ± 0.00 |
| Proteobacteria | *Vibrionaceae* | ***Vibrio* sp.** | 0.00 ± 0.00 | 0.00 ± 0.00 | 0.00 ± 0.00 | 0.00 ± 0.00 | 0.07 ± 0.01 | 0.00 ± 0.00 |
| Actinobacteriota | *Nocardiaceae* | ***Nocardia* sp.** | 0.00 ± 0.00 | 0.00 ± 0.00 | 0.00 ± 0.00 | 0.00 ± 0.00 | 0.07 ± 0.01 | 0.00 ± 0.00 |
| Bacteroidota | env.OPS 17 | **env.OPS 17 sp.** | 0.00 ± 0.00 | 0.00 ± 0.00 | 0.00 ± 0.00 | 0.00 ± 0.00 | 0.07 ± 0.01 | 0.04 ± 0.00 |
| Planctomycetota | Order  Planctomycetales | **Order  Planctomycetales** | 0.00 ± 0.00 | 0.00 ± 0.00 | 0.00 ± 0.00 | 0.00 ± 0.00 | 0.03 ± 0.00 | 0.07 ± 0.00 |
| Verrucomicrobiota | cvE6 | **Chlamydiales bacterium** | 0.00 ± 0.00 | 0.00 ± 0.00 | 0.00 ± 0.00 | 0.00 ± 0.00 | 0.01 ± 0.00 | 0.00 ± 0.00 |
| Planctomycetota | *Gemmataceae* | ***Gemmataceae*** | 0.00 ± 0.00 | 0.00 ± 0.00 | 0.00 ± 0.00 | 0.00 ± 0.00 | 0.01 ± 0.00 | 0.02 ± 0.00 |
| Proteobacteria | *Halieaceae* | **OM60(NOR5) clade sp.** | 0.00 ± 0.00 | 0.00 ± 0.00 | 0.00 ± 0.00 | 0.00 ± 0.00 | 0.01 ± 0.00 | 0.00 ± 0.00 |
| MBNT15 | MBNT15 | **MBNT15 sp.** | 0.00 ± 0.00 | 0.00 ± 0.00 | 0.00 ± 0.00 | 0.00 ± 0.00 | 0.01 ± 0.00 | 0.00 ± 0.00 |
| Actinobacteriota | *Microbacteriaceae* | ***Microbacterium* sp.** | 0.00 ± 0.00 | 0.00 ± 0.00 | 0.00 ± 0.00 | 0.00 ± 0.00 | 0.00 ± 0.00 | 0.03 ± 0.00 |
| Actinobacteriota | 67-14 | **67-14 sp.** | 0.00 ± 0.00 | 0.00 ± 0.00 | 0.00 ± 0.00 | 0.00 ± 0.00 | 0.00 ± 0.00 | 0.03 ± 0.00 |
| Cyanobacteria | *Gastranaerophilales* | ***Gastranaerophilales* sp.** | 0.00 ± 0.00 | 0.00 ± 0.00 | 0.00 ± 0.00 | 0.00 ± 0.00 | 0.00 ± 0.00 | 0.02 ± 0.00 |
| Proteobacteria | *Rhizobiales Incertae Sedis* | ***Phreatobacter* sp.** | 0.00 ± 0.00 | 0.00 ± 0.00 | 0.00 ± 0.00 | 0.00 ± 0.00 | 0.00 ± 0.00 | 0.02 ± 0.00 |
| Actinobacteriota | *Nocardiaceae* | ***Rhodococcus* sp.** | 0.00 ± 0.00 | 0.00 ± 0.00 | 0.00 ± 0.00 | 0.00 ± 0.00 | 0.00 ± 0.00 | 0.02 ± 0.00 |
| Verrucomicrobiota | *Verrucomicrobiaceae* | ***Prosthecobacter* sp.** | 0.00 ± 0.00 | 0.00 ± 0.00 | 0.00 ± 0.00 | 0.00 ± 0.00 | 0.00 ± 0.00 | 0.01 ± 0.00 |
| Firmicutes | *Paenibacillaceae* | ***Paenibacillus* sp.** | 0.00 ± 0.00 | 0.00 ± 0.00 | 0.00 ± 0.00 | 0.00 ± 0.00 | 0.00 ± 0.00 | 0.01 ± 0.00 |
| Proteobacteria | *Rhodobacteraceae* | ***Rhodobacteraceae*** | 0.00 ± 0.00 | 0.00 ± 0.00 | 0.00 ± 0.00 | 0.00 ± 0.00 | 0.00 ± 0.00 | 0.01 ± 0.00 |
| Actinobacteriota | *Solirubrobacteraceae* | ***Conexibacter* sp.** | 0.00 ± 0.00 | 0.00 ± 0.00 | 0.00 ± 0.00 | 0.00 ± 0.00 | 0.00 ± 0.00 | 0.01 ± 0.00 |
| Actinobacteriota | *Iamiaceae* | ***Iamia* sp.** | 0.00 ± 0.00 | 0.00 ± 0.00 | 0.00 ± 0.00 | 0.00 ± 0.00 | 0.00 ± 0.00 | 0.01 ± 0.00 |
